# Evaluation of single-template ligand-based methods for the discovery of small-molecule nucleic acid binders

**DOI:** 10.1093/bib/bbaf620

**Published:** 2025-11-21

**Authors:** Dávid Bajusz, Anita Rácz, Janusz M Bujnicki, Filip Stefaniak

**Affiliations:** Medicinal Chemistry Research Group, Hungarian Research Network Research Centre for Natural Sciences, Magyar tudósok krt. 2, Budapest 1117, Hungary; Plasma Chemistry Research Group, Hungarian Research Network Research Centre for Natural Sciences, Magyar tudósok krt. 2 Budapest 1117, Hungary; Laboratory of Bioinformatics and Protein Engineering, International Institute of Molecular and Cell Biology in Warsaw, ul. Ks. Trojdena 4, Warsaw, 02-109, Poland; Laboratory of Bioinformatics and Protein Engineering, International Institute of Molecular and Cell Biology in Warsaw, ul. Ks. Trojdena 4, Warsaw, 02-109, Poland

**Keywords:** RNA, DNA, nucleic acids, RNA-ligand interactions, molecular fingerprints, similarity

## Abstract

Nucleic acid molecules, including ribonucleic acid (RNA) and deoxyribonucleic acid (DNA), are essential for various biological processes and can adopt diverse 3D structures that serve as potential drug targets. The direct targeting of nucleic acid structures by small molecules represents an emerging field in drug design with enormous potential for providing therapeutic options for diseases that are currently not addressed, including genetic diseases and viral infections. In the early days of this promising field, a shortage of reliable structural data presents a bottleneck to the direct adaptation of structure-based methods, making the simpler yet powerful ligand-based approach an attractive alternative for virtual screening. In this study, we thoroughly evaluate and benchmark these methods against the reported binding of small molecules to diverse nucleic acid targets. We also compare these methods with structure-based molecular docking. Our results demonstrate that classification performance is significantly influenced by the applied descriptors, the chosen similarity measure, and the specific nucleic acid target. We have also proposed a consensus method that combines the best-performing algorithms of distinct nature. According to our studies, this approach outperforms all other tested methods, providing a valuable framework for nucleic acid-targeted drug discovery.

## Introduction

Ribonucleic acid (RNA) molecules are essential for various biological processes, including the transmission of genetic information, the response to cellular signals, and the catalysis of chemical reactions [1]. RNA structures and functions are often modulated by small chemical molecules, including both naturally occurring molecules and those obtained through synthetic organic chemistry. Many RNA molecules, such as bacterial ribosomes and riboswitches, are known or predicted targets of small-molecule drugs [2]. The continuous discovery of new functional RNAs involved in various biomedically important processes increases the demand for the development of new small molecules targeting RNA, and for new methods for analyzing RNA-small molecule ligand interactions. The recent approval of a small-molecule regulator of RNA splicing, Risdiplam, for the treatment of spinal muscular atrophy showcases the significant therapeutic potential of targeting RNA with small molecules [3]. This emerging paradigm emphasizes the need to further explore the applicability of chemo- and bioinformatics methods to the development of new ligands that bind to RNA.

Interactions between small molecules and deoxyribonucleic acid (DNA) also play a crucial role in numerous biological processes and represent important targets for therapeutic intervention. DNA-binding molecules can modulate gene expression by interfering with transcription factor binding, altering DNA replication and repair mechanisms, or inducing structural changes that affect chromatin organization. Although DNA typically adopts the standard B-DNA conformation, it can also form alternative structures such as hairpins, Holliday junctions, cruciforms, triplexes, and G-quadruplexes, which can accommodate small-molecule ligands [4, 5].

Computational drug design approaches are usually grouped into structure-based and ligand-based alternatives, and as the name suggests, ligand-based methods rely purely on information about the small molecules being investigated, without any explicit structural input from the target side. Molecular similarity calculations are a cornerstone of cheminformatics [6], and for a long time, they were central tools in virtual screening, particularly for targets with unknown structures [7]. Nowadays, with the advent of high-throughput, accurate protein modeling methods [8], and the rapid growth of the accessible (generated or on-demand) chemical space [9], similarity-based methods are typically used as the first stage of screening to reduce large datasets to a size manageable by more sophisticated structure-based approaches. Their speed and interpretability also make them suitable components of more complex, artificial intelligence (AI)-assisted workflows [10]. While there is a wide range of available choices both for the fingerprints (0/1 or count-based encodings of molecular features) used for representing the molecules, and for the similarity measures that are used for comparing and quantifying the similarities of such fingerprints [11], some popular choices have emerged over time, including MACCS keys [12] and extended connectivity fingerprints (ECFP [13]) in the fingerprint space, and the Tanimoto coefficient as the most universally applied similarity measure [14]. Nonetheless, our recent works have shown that the optimal selection is far from trivial, so a case-by-case investigation can yield significantly better combinations than just selecting the most popular choices [15, 16]. Besides fingerprint similarity, 3D methods are also of great importance in ligand-based virtual screening, with prominent examples being small molecule superposition using a suitable spatial representation such as shape or Gaussian volume [17], and pharmacophore screening, which projects the molecules onto 3D point sets, a greatly simplified spatial representation that can also encapsulate some information on the key interacting features required for on-target effect [18]. From the many open-source ligand-based methods, LiSiCA employs a maximum clique algorithm on molecular graphs with atom-level representation to identify common substructures and calculate similarity scores based on both topology and spatial arrangement [19]. SHAFTS combines shape-based alignment with pharmacophore feature matching through a feature triplet hashing method, enabling rapid enumeration of alignment poses followed by optimization of Gaussian volume overlap [20]. ShaEP uses field-graph matching based on electrostatic potential and local shape descriptors at molecular surfaces, subsequently optimizing alignments through volume overlap maximization [21]. Align-It (originally, Pharao) likewise adopts a hybrid approach, representing pharmacophore features as Gaussian volumes to align molecular graphs [22].".

While the application of ligand-based methods is an everyday routine for conventional protein targets, this rich toolbox is still relatively underexplored in the emerging field of DNA- and RNA-binding small molecules. There are not many studies showing the application or benchmarking of ligand-based methods for the discovery of RNA-binding small molecules. Parkesh *et al.* describe the application of 3D shape similarity and the ROCS software (Rapid Overlay of Chemical Structures, OpenEye Scientific Software, Inc., Santa Fe, NM, USA, http://www.eyesopen.com/documentation) in the search for small molecules targeting r(CUG)12-MBNL1 complex formation, associated with Myotonic dystrophy type 1 disorder [23]. The search was done using two templates—Pentamidine and Hoechst 33258, with further diversification and visual inspection of potential hits. *In vitro* tests indicated 17 molecules that are more potent than the compounds used as templates. Morgan *et al.* conducted an analysis of cheminformatic and shape-based descriptors for 104 biologically active RNA-targeted ligands from the RNA-targeted BIoactive ligaNd Database (R-BIND) [24]. Comparison of these molecules with the reference groups showed the importance of the shape of the molecules—captured by shape-based descriptors—for activity. Molecular fingerprints have been reported as inputs to machine learning algorithms to build predictive models [25, 26], but in these cases a number of active/binding molecules are required. Furthermore, when using an ML classifier, one needs to provide not only positive examples of ligands (actives) but also negative examples (inactives). With very limited screening data available for RNA targets, these requirements can be significantly constraining for model development.

In the meantime, structure-based approaches are gaining a foothold in RNA-targeted drug discovery, as well. Open-access datasets like HARIBOSS for RNA-ligand structures [27], or ROBIN for small-molecule ligands annotated with RNA-bioactivities [25] provide strong starting points for virtual screening campaigns, while experimental approaches like FOREST (folded RNA element profiling with structure library) are accelerating the high-throughput discovery of folded RNA elements and their RNA-protein interaction networks [28]. Still, the availability of experimentally solved 3D structures of RNA is very scarce compared to protein targets (1000 structures collected in HARIBOSS versus >240 000 structures in the PDB), and the accuracy of modeling methods may still be a limiting factor for the structure-based methods [29, 30]. This fact, in connection with the growing interest in the role of RNA in multiple diseases, motivates the development and validation of computational approaches that are less dependent on structural information. Ligand-based methods offer a promising direction for RNA drug discovery, potentially circumventing the structural data limitations while accelerating the identification of novel therapeutic candidates targeting disease-relevant RNA molecules.

The assumption of the underlying principle of molecular recognition (shape/feature complementarity) is the same for protein-ligand and DNA/RNA-ligand interactions, which means that the methodologies that were developed for the protein-ligand complexes could be directly applied for the DNA/RNA-ligand systems. However, the fundamental difference between the molecular building blocks of proteins (20 amino acids) and those of DNA or RNA (4 nucleobases) has far-reaching implications for the higher-order structure of these macromolecules, and for the influence of small-molecule binders. Therefore, a detailed investigation and comparison of the available methods is necessary for understanding their applicability in this exciting field. Here, we collect, compare and benchmark 16 fingerprints and 6 other, single-template, ligand-based virtual screening methods against a wide selection of DNA/RNA-ligand datasets, with the aim to provide a roadmap for prospective applications. An extension of the analysis to multi-template and consensus methods is planned in a follow-up effort.

## Materials and methods

Small molecule dataset and binding data were taken from Yazdani *et al.* [25]. Molecular structures were standardized using the RDKit Normalizer in KNIME (version 4.7.8) [31]. Fingerprints and descriptors were calculated using in-house developed python programs, KNIME, or dedicated tools (Table 1). Similarity metrics (Table 2) were calculated using RDKit (version 2024.03.5). Data analysis and visualization were performed using Jupyter notebooks with matplotlib and seaborn modules. Vector similarity was calculated as the Manhattan or Euclidean distance between RDKit or Spectrophores [38] descriptor vectors and then converted to similarity. Consensus scores were calculated as the average or maximum (abbreviations: avg and max, respectively) scores from the indicated pool of scores for a single molecule.

**Table 1:** Fingerprints and other ligand-based virtual screening methods used for benchmarking.

Abbreviation	Description	Calculation method
Random scores	Randomly generated scores	Python
Fingerprints		
FP: random bits (512, 10%)	Bit vector of length 512 bits and density 10%, with bits set randomly.	Python
FP: CDK ECFP*n*	CDK extended-connectivity fingerprint *n*, *n* = {0, 2, 4}	KNIME (4.7.8) + CDK [32, 33]
FP: CDK FCFP*n*	CDK functional-connectivity fingerprint *n*, *n* = {0, 2, 4}	
FP: CDK Estate	CDK estate fingerprint	
FP: CDK MACCS	CDK MACCS fingerprint	
FP: CDK PubChem	CDK PubChem fingerprint	
FP: CDK standard	CDK standard fingerprint	
FP: indigo	Indigo fingerprint	KNIME + Indigo [34]
FP: MAP4 (*n* bits)	MAP4 (*n* bits) fingerprint, *n* = {256, 512, 1024, 2048}	MAP4 [35]
FP: OpenBabel FP*n*	OpenBabel FP*n* fingerprint; *n* = {2, 3, 4}	Python + OpenBabel [36]
FP: RDKit AtomPair	RDKit Atom pair fingerprint	KNIME + RDKit [37]
FP: RDKit Avalon	RDKit Avalon fingerprint	
FP: RDKit FeatAtom Morgan	RDKit features Atom Morgan fingerprint	
FP: RDKit layered	RDKit layered fingerprint	
FP: RDKit Morgan	RDKit Morgan fingerprint (ECFP-like)	
FP: RDKit	RDKit fingerprint	
FP: RDKit torsion	RDKit topological torsion fingerprint	
Other ligand-based methods		
KNIME 3D shape similarity	KNIME 3D shape similarity	KNIME
LISICA {2D, 3D}	LISICA similarity calculated using 2D and 3D features	LISICA (1.0.1) [19]
Align-It (score type)	Align-It: Tanimoto similarity; Tversky similarity: the “reference” measure is primarily intended to identify database compounds that have a pharmacophore which is a superset of the reference pharmacophore; the “DB” measure is useful for identifying database compounds that have a pharmacophore which is a subset of the reference pharmacophore.	Align-it (1.0.3) [22]
SHAFTS (score type)	SHAFTS: feature, shape, and hybrid score	SHAFTS (2011) [20]
ShaEP (score type)	ShaEP: shape, ESP, average, and best score	ShaEP (1.4.0.0) [21]
Vect. Sim. (method) descriptors	Similarity of vectors of “descriptors” calculated using the “method”. Descriptors: RDKit descriptors (2D) [37] Spectrophores (3D) [38]	Python

**Table 2:** Similarity measures tested for fingerprint and descriptor vector similarity.

Similarity measure	Description	Definition
Braun-Blanquet	A coefficient used to compare the presence of attributes between sets.	c/max(a,b)
Tanimoto	Also known as the Jaccard Index, it measures the similarity between two sets by dividing the size of the intersection by the size of the union.	c/(a + b-c)
Kulczynski	A measure of similarity that is the average of two ratios: the number of shared attributes to the total number of attributes in each set.	0.5 ^*^ (c/a + c/b)
Cosine	Measures the cosine of the angle between two non-zero vectors, often used in text and molecular similarity.	c/sqrt(a^*^b)
Russell-Rao	A binary similarity measure that considers only the positive matches between vectors.	c/n
Tversky (query- and reference-weighted)	A generalization of the Tanimoto coefficient, measuring the similarity between two sets with an asymmetric weight, favoring one of the objects (molecules). Here, the reference-weighted version (α = 0.3, β = 0.7) emphasizes features present in the template molecule, while the query-weighted version (α = 0.7, β = 0.3) emphasizes features in the query molecule.	c/(α *a + β* b) ^*^ Query-weighted (α = 0.7, β = 0.3) ^*^ Reference-weighted (α = 0.3, β = 0.7)
Euclidean	Euclidean distance *d* between two vectors of descriptors (u,v) is the straight-line distance between the points in Euclidean space. Converted to similarity *sim* for consistency.	d(u,v) = √((Σ((u_i - v_i)^2^)) sim(u,v) = 1 / (1 + d(u,v))
Manhattan	Manhattan distance *d* between two vectors of descriptors (u,v) is the sum of the absolute differences between corresponding elements of the vectors. Converted to similarity *sim* for consistency.	d(u,v) = Σ(|u_i - v_i|) sim(u,v) = 1 / (1 + d(u,v))

Where: a = number of bits set in the first fingerprint, b = number of bits set in the second fingerprint, c = number of bits common to both fingerprints, *n* = total number of bits in the fingerprint.

FP3 fingerprint analysis was performed to identify the chemical features that distinguish RNA *binders* from *non-binders* across all 55 bit positions. To compare the mean bit frequencies between the groups, we used independent t-tests for statistical significance testing. We also used Mann–Whitney U tests and chi-square tests as additional validation methods. All of these were implemented using SciPy statistical functions. Multiple testing correction was applied using the Bonferroni method (the statsmodels Python module) to account for the simultaneous testing of all 55 bits. Effect sizes were quantified using Cohen’s d to evaluate practical significance beyond statistical significance. Feature importance was assessed using mutual information scores, calculated with scikit-learn’s mutual_info_classif function (estimation of mutual information for a discrete target variable), as well as chi-square feature selection scores and Pearson correlation coefficients.

Fingerprint similarity was calculated in Python using DataStructs.cDataStructs module of RDKit, while Euclidean and Manhattan distances between descriptor vectors were calculated using the metrics.pairwise module of Scikit-Learn [39] and converted to similarity (see Table 2 for details). Statistical tests (t-test, Mann–Whitney U test, and Mood’s median test), were performed using SciPy [40], and two-tailed *p*-values are reported. Areas under the ROC curve and further performance metrics were calculated as described in our previous work [41]. For target-specific performance visualization (Fig. 4), we generated pooled ROC curves by concatenating similarity scores from all template molecules within each target, thereby providing an aggregate assessment of each method’s discriminative ability for that particular nucleic acid target.

For the molecular docking experiment, we used the experimentally solved structure of HBV (PDB ID: 6VAR, pdb_00006var, the first model). Computational models of PreQ1 and ZTP were derived using Protenix web server, with default options and the protenix_base_default_v0.5.0 model [42]. Molecular docking was performed using rDock docking program [43]. Binding pockets were predicted using DRLiPS method [44]. 3D conformations of ligands were generated using OpenBabel.

## Results and discussion

For the benchmark we used data from the Repository Of BInders to Nucleic acids (ROBIN) described by Yazdani *et al.* [25]. It was derived from screening 24,572 commercially available drug-like small molecules against 36 nucleic acid targets (27 RNA and 9 DNA structures) representing different structural classes such as hairpins, G-quadruplexes, pseudoknots, three-way junctions and triple helices. The screen yielded a total of 1,627,072 data points and provided binary binding data (*binder* or *non-binder*). The library included compounds from several commercial sources. This screening identified 2,188 unique small molecules as hits for at least one nucleic acid target, including 2,003 that specifically bound to RNA targets. Hit rates for all structural classes were <1%, comparable to typical hit rates for protein targets in unbiased screening efforts [9]. It is important to note that each target had a unique set of hits, so target selectivity was not investigated in the present work.

### Fingerprint-based methods and similarity measures

Molecular fingerprints are compact representations of chemical structures that encode structural features in binary or numerical vectors. These descriptors are widely used in cheminformatics for comparing compounds and predicting their properties without relying on 3D structural information.

In our evaluation of ligand-based methods for nucleic acid target screening, we systematically investigated different fingerprint types and similarity measures. We used a diverse set of fingerprints including extended connectivity fingerprints (ECFP0, ECFP2, ECFP4), functional connectivity fingerprints (FCFP0, FCFP2, FCFP4), traditional substructure key-based fingerprints (MACCS keys, PubChem fingerprints, OpenBabel FP3 and FP4), as well as other fingerprint types like E-state fingerprints that are based on the E-state topological index of Hall and Kier [45]. Each fingerprint type captures different aspects of molecular structure, from simple substructure patterns to more complex connectivity environments. While extended connectivity fingerprints are quite widely used [13], other representations might provide better performance depending on the considered dataset. For instance, further abstraction can be achieved by functional classes (similar to pharmacophore features) in FCFP fingerprints [11], but the usage of orthogonal concepts like the E-state index or topological torsions [46] might be beneficial as well.

To quantify molecular similarity, we used seven different similarity metrics, including the Braun-Blanquet, Cosine, Kulczynski, Russell-Rao, Tanimoto and Tversky (Query and Reference weighted) coefficients, as implemented in the RDKit cheminformatics package. These metrics differ in how they weigh shared and unshared features, potentially affecting their ability to identify biologically relevant similarities for nucleic acid binding compounds. In our earlier work, we performed a thorough virtual screening benchmark of similarity measures for protein targets [15], but to our knowledge, no comparable benchmarking study has been conducted for nucleic acids.

**Figure 1:**
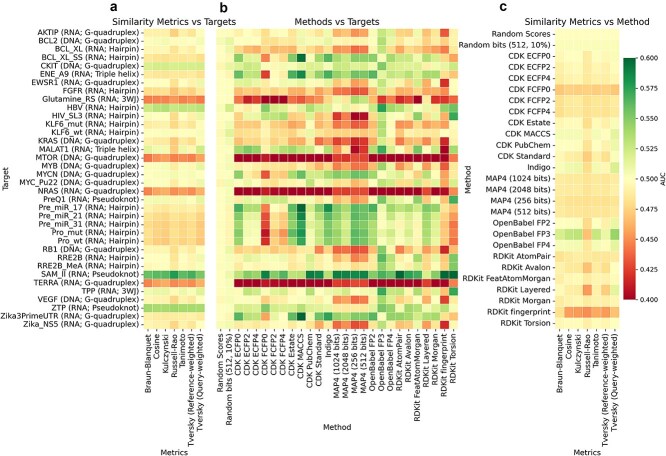
Heatmaps of mean AUC values as a function of (a) the applied similarity metric (X axis) versus the examined dataset (Y axis), (b) the applied fingerprint (X axis) versus the examined dataset (Y axis), and (c) the applied similarity metric (X axis) versus the applied fingerprint (Y axis). Molecular target names are followed by the type of nucleic acid (RNA or DNA) and the structural class.

To define performance baselines, we introduced two negative controls: “Random Scores”, which assigned random similarity values unrelated to molecular structure, and “FP: random bits” consisting of bit vectors of length 512 and 10% density. These controls provided reference points representing the absence of predictive power, with expected area under the curve (AUC) values close to 0.50, characteristic of random classification. Random fingerprints generated with further combinations of length (512, 1024, 2048) and density (1%, 5%, 10%) have shown essentially the same performances (data not shown). Results are summarized in Fig. 1 and values are provided in Table S6. In Fig. 1a, in agreement with the results on each factor alone, there are more prominent bands in the horizontal direction, i.e. stronger outliers can be identified among the datasets than the similarity metrics. Interestingly, the otherwise worst performing similarity metric, Russell-Rao, gives the best result for the best-classified SAM_II dataset. From the fingerprints, the OpenBabel fingerprints (particularly FP3) stand out, particularly when combined with the Tversky (Query-weighted) similarity measure.

Among the fingerprint-based methods, the OpenBabel FP3 fingerprint showed superior performance (mean AUC for all targets equal to 0.551), outperforming other fingerprint types, including the widely used ECFP variants. Interestingly, within the ECFP family we observed a consistent decrease in performance with increasing radius parameters, with ECFP0 (AUC = 0.509) outperforming ECFP2 (AUC = 0.508) and ECFP4 (AUC = 0.503). This trend contrasts with virtual protein-ligand screening, where larger radii generally confer superior screening performance [47]. The difference in performance suggests that local atomic environments represented in simpler fingerprints may better encode the structural features relevant to nucleic acid binding.

An important observation is the notably strong performance of the OpenBabel FP3 fingerprint compared to other fingerprint types. While modern circular fingerprints like ECFPs are generally favored in protein-ligand screening campaigns, the FP3 fingerprint—based on SMARTS patterns that encode specific functional groups and structural motifs—demonstrated marked superiority for nucleic acid targets. This suggests that RNA/DNA binding may be more dependent on the presence of particular structural elements rather than complex atomic environments captured by circular fingerprints. The structural key-based approach of FP3, which explicitly encodes hydrogen bonding patterns, aromatic features, and specific ring systems, appears to more effectively capture the interaction patterns relevant for nucleic acid recognition. This finding challenges the conventional wisdom that more complex fingerprints invariably yield better performance and indicates that the choice of molecular representation should be carefully tailored to the specific biomolecular target class.

Other notable fingerprinting methods included OpenBabel FP4 (AUC = 0.522) and Indigo fingerprints (AUC = 0.522), both of which achieved statistically significant improvements over random classification (*p* < .0001).

**Figure 2:**
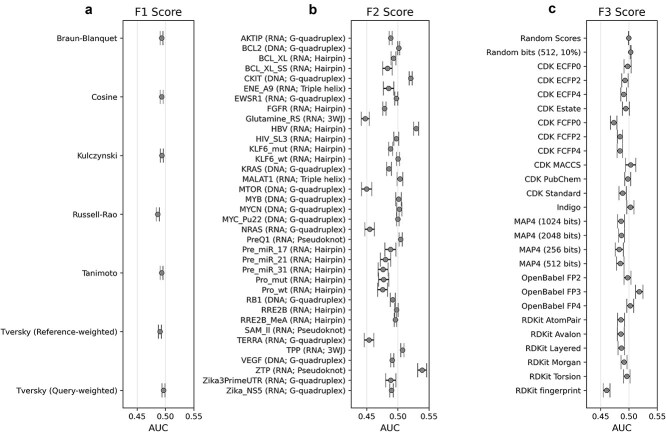
Effects of the different factors on classification performance: similarity metric (panel a, factor F1), target/dataset (panel b, factor F2), and fingerprint (panel c, factor F3). Molecular target names are followed by the type of nucleic acid (RNA or DNA) and the structural class.

In the case of fingerprint-based similarity methods, we used analysis of variance (ANOVA) to dissect the effects of several factors upon classification performance, i.e. the resulting AUC values, following the methodology in our earlier works. Chiefly, the effects of the applied similarity metric (7 levels), the considered dataset (36 levels), and the applied fingerprints (45 levels) were considered as factors (Fig. 2). While there is generally a minor effect of the chosen similarity metric, we can identify the Query-weighted Tversky coefficient to be significantly better, and the Russell-Rao metric to be significantly worse than the benchmark level defined by the other metrics. By comparison, the effect of the dataset considered is much more significant: here, we can identify positive (e.g. SAM_II) and negative (e.g. Glutamine_RS) outliers as well. Based on the width of the confidence bands, we can identify cases that are less sensitive (e.g. BCL2, RRE2B_MeA) or more sensitive (e.g. BCL_XL_SS or Pre_miR_^*^ or Pro_^*^) to the particular choice of fingerprint/similarity methods (Fig. 2b). The effect of the applied fingerprint method is, once again, quite significant, with both positive (e.g. Openbabel FP3) and negative outliers (e.g. cdk_FCFP0 or the RDKit fingerprint). Interestingly, the performance of the popular class of ECFP fingerprints deteriorates with increasing radius (Fig. 2c).

Our comprehensive analysis of fingerprint-based methods for nucleic acid targets reveals striking differences in performance between fingerprint types and similarity metrics. To our surprise, a relatively simple fingerprint—the OpenBabel FP3—emerged as the best performing fingerprint, with median AUC values consistently >0.54 for most metrics, reaching 0.569 for the Query-weighted Tversky metric. This combination clearly outperforms the other fingerprints, with OpenBabel FP4 as the second-best performing fingerprint (median AUC ~0.51–0.52). Both top-performing fingerprints are substructure key-based, relying on a set of SMARTS patterns, with 55 and 307 bits for FP3 and FP4, respectively. This suggests that the relatively simple set of chemical patterns can help discriminate between binding and non-binding molecules, possibly due to their representation of hydrogen bonding patterns and aromatic systems prevalent in nucleic acid-ligand interactions. Indeed, this hypothesis was corroborated by a statistical analysis of FP3 fingerprints across all molecules in our dataset, which highlighted significant frequency differences and elevated feature importance scores for several bit positions that describe hydrogen-bonding moieties (Supplementary Note 1).

The choice of similarity metric proved crucial, with Query-weighted Tversky consistently giving the best results for most fingerprints. This asymmetric similarity calculation, which gives greater weight to features present in the query molecule, appears to be particularly suitable for predicting nucleic acid binding. Conversely, the Russell-Rao metric performed poorly for most fingerprints, probably because it focuses only on positive matches and ignores both the disagreeing features, as well as the common absence of features. Traditional metrics such as Tanimoto and Cosine performed similarly, suggesting that the choice of fingerprint has a greater impact on performance than the choice between these more common similarity coefficients.

Several notable trends in fingerprint design emerged. For CDK ECFPs, lower radius values (ECFP0) outperformed higher radii (ECFP2, ECFP4), suggesting that local chemical features may be more informative than extended connectivity patterns for nucleic acid binding. Similarly, atom-based fingerprints generally outperformed their feature-based counterparts (ECFPs versus FCFPs).

MAP4 fingerprints and the RDKit base fingerprint consistently underperformed, with median AUC values below the random baseline (0.5), suggesting that these methods may not be suitable for nucleic acid targets, despite their utility in protein-ligand screening [48]. These observations highlight that fingerprinting methods optimized for protein targets may require different design considerations for nucleic acid applications. The clear performance hierarchy observed among fingerprints and metrics provides valuable guidance for computational chemists seeking to apply ligand-based virtual screening to nucleic acid targets, with OpenBabel FP3 combined with Query-weighted Tversky similarity offering the most promising standalone fingerprint approach.

### Other ligand-based methods

**Figure 3:**
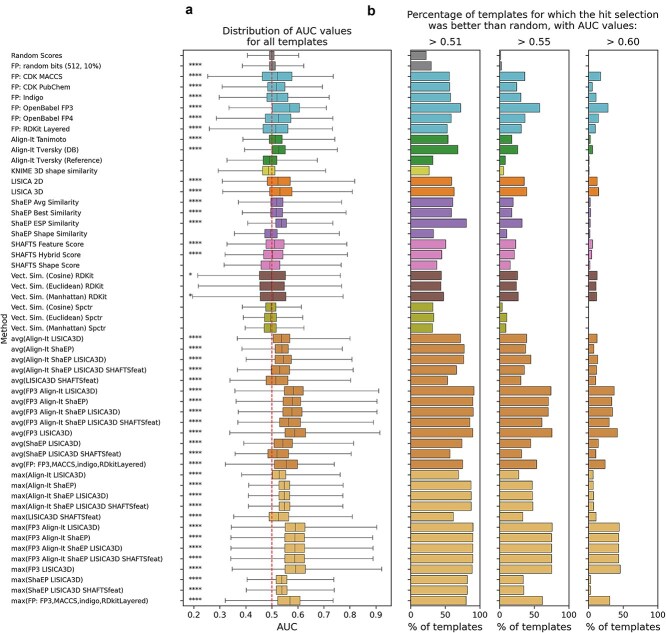
Comparison of the performance for ligand-based methods for all nucleic acid targets. (a) The distribution of the AUC values for all templates used for analysis. The dashed vertical line at AUC = 0.50 indicates the baseline for classification performance. Statistical significance was assessed using a paired *T*-test, comparing each method to the “random scores” (negative control). The significance levels are shown next to each boxplot for methods for which the mean AUC value was higher than for “random scores” (AUC = 0.500) and are denoted by asterisks (^*^P < .05, ^**^P < .01, ^***^P < .001, ^*****^P < .0001). (b) Percentage of molecular templates (for all molecular targets) where the AUC values were >0.50 (0.51, 0.55, and 0.60). For fingerprint-based methods, the query-weighted Tversky similarity measure was used. Molecular target names are followed by the type of nucleic acid (RNA or DNA) and the structural class.

We then evaluated the performance of different ligand-based methods in predicting the binding of small molecules to nucleic acid targets. Our comparison included well-established approaches: shape-based (KNIME 3D shape similarity, ShaEP, and SHAFTS), pharmacophore-based (Align-it), feature-based (LISICA) and methods that compare vectors of properties (spectrophores and simple molecular descriptors). While these approaches have demonstrated utility in virtual screening for protein targets, their accuracy for nucleic acids has not been systematically evaluated. In the comparison, we also included the previously selected best-performing fingerprinting methods (with the best-performing Query-weighted Tversky similarity measure) to provide a complete view of the performance landscape (see Fig. 3 for AUC values and Fig. S1 for further performance metrics).

Among the ligand-based methods, the best-performing choices include LISICA (3D variant), ShaEP (with ESP similarity) and Align-It (Tversky DB) with AUC values of 0.536, 0.536 and 0.531, respectively. The higher performance of these methods supports the importance of accounting for both the presence of specific chemical features and their 3D arrangement when modeling RNA/DNA-ligand interactions. This is consistent with previous observations that shape complementarity is a critical factor in RNA-targeted ligand binding, as noted by Morgan *et al.* in their analysis of the R-BIND database. However, on average these are slightly worse than the performance of the FP3 fingerprint discussed above (mean AUC = 0.551). To note, almost all methods provide significantly better results than random scoring, as established by paired t-tests of the AUC value distributions. Likewise, AUC distributions for other similarity metrics versus Tanimoto are significant at varying levels (except for Braun-Blanquet), highlighting the benefit of considering alternative metrics beyond the most popular choice (Supplementary Fig. S7). It is also important to note that while the average AUC values might just be over random (0.5), for each method we can find at least a small number of ligand templates (i.e. reference molecules) that provide significantly better-than-random classification/screening performances (Fig. 3b).

Consensus approaches, which combine multiple scoring methods, consistently delivered the best overall performance in our benchmark. The best performing method across all categories was the combination of FP3 and LISICA3D scores (both avg and max), which achieved a mean AUC of 0.595. This consensus approach effectively integrates two-dimensional structural information with 3D molecular recognition features. When comparing the average-based and maximum-based consensus strategies, we found only marginal differences in their overall performance (mean AUCs values for all consensus methods equal 0.565 for both cases), suggesting that both approaches are suitable for combining individual scoring methods. The substantial improvement of the consensus methods over their individual components (e.g. a 0.044 improvement in AUC for avg(FP3 LISICA3D) compared to its best component) underlines the complementary nature of different similarity measures in capturing different aspects of molecular recognition. These results demonstrate that the judicious combination of fingerprint-based methods with 3D approaches allows for a more comprehensive assessment of potential RNA/DNA-binders, providing a practical strategy for improving the effectiveness of virtual screening for these challenging targets.

Another notable finding is that this minimal combination of just two complementary methods—integrating 2D structural keys (FP3) with 3D pharmacophore information (LISICA3D)—performs almost as well as more complex consensus approaches involving additional methods. For example, the combination of four methods in avg(FP3 Align-It ShaEP LISICA3D) yielded an AUC of 0.585, only marginally better than the simpler two-method consensus. This suggests that most performance gains can be achieved by combining only a few complementary methods, with additional components yielding diminishing returns. This finding has important practical implications for computational screening workflows, as it suggests that researchers can optimize both performance and computational efficiency by focusing on smaller, well-chosen method combinations rather than more complex consensus schemes. It also mirrors the conclusions of Svensson *et al.* in their evaluation of consensus approaches in virtual screening against protein targets [49].

**Figure 4:**
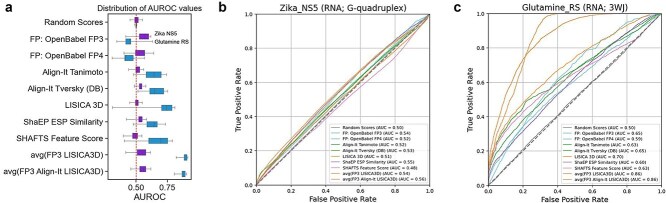
The performance of the selected methods for two RNA targets: Zika NS5 and glutamine riboswitch: (a) Distribution of AUROC values; (b) and (c) Pooled ROC curves (predicted probabilities and active/inactive labels concatenated from all reference ligands).

In both cases, performance is heavily target-dependent, as illustrated by two examples in the bar plots in Fig. 4. Here, we can observe that Openbabel fingerprints perform better on the Zika_NS5 dataset, while other methods perform better on the Glutamine_RS dataset. It is reasonable to expect that for Zika_NS5 ligands, common structural features that are efficiently captured by OpenBabel fingerprints are important for binding affinity. At the same time, Glutamine_RS ligands are not captured so efficiently by fingerprint-encoded features, but rather by analogies in shape or pharmacophoric composition. The distribution of AUC values for individual targets is shown in Fig. S2.

Analysis of performance across nucleic acid types revealed minor differences, with RNA targets showing slightly higher mean and median AUC values compared to DNA targets (Fig. S3). Similarly, we observed modest performance variations among different structural motif types, though these differences were not substantial (Fig. S4). Notably, we found no correlation between method performance and nucleic acid target size (expressed as the number of nucleotides, Fig. S5).

We investigated the influence of 3D methods on screening performance (Fig. S6 and Table S8). Direct comparison of the LISICA algorithm in its 2D and 3D versions revealed only marginal improvement for the latter: AUC increased by 0.007 (1.3% relative increase) and BEDROC by 0.002 (2.4% increase). This suggests that 3D conformational information alone provides limited benefits when used in isolation. More striking, however, is the synergistic enhancement observed when combining 3D methods with other approaches. While Align-It alone yielded an average AUC of 0.531, combining it with another 3D method (LISICA 3D) increased the AUC to 0.541 (+1.9%), and the three-way consensus with LISICA 3D and ShaEP further improved performance to AUC = 0.546 (+2.8%). These results indicate that incorporating multiple sources of 3D structural information enhances the performance of consensus methods. Similar improvements were observed for the FP3 fingerprint when combined with 3D methods: FP3 alone achieved an AUC of 0.551, which increased to 0.577 (+4.7%) when paired with LISICA 3D, and to 0.574 (+4.2%) in a three-way consensus with LISICA 3D and Align-It. Notably, all early-enrichment metrics showed even more substantial improvements in these consensus approaches, with some combinations exhibiting >100% relative increase in EF_1%_ compared to individual methods. While individual 3D methods show limited standalone improvement, their real value emerges in consensus approaches, where 3D information provides complementary perspectives to 2D topological descriptors. This supports the use of ensemble methods that integrate both 2D and 3D molecular representations into a unified consensus scoring framework.

### Scores distribution

**Figure 5:**
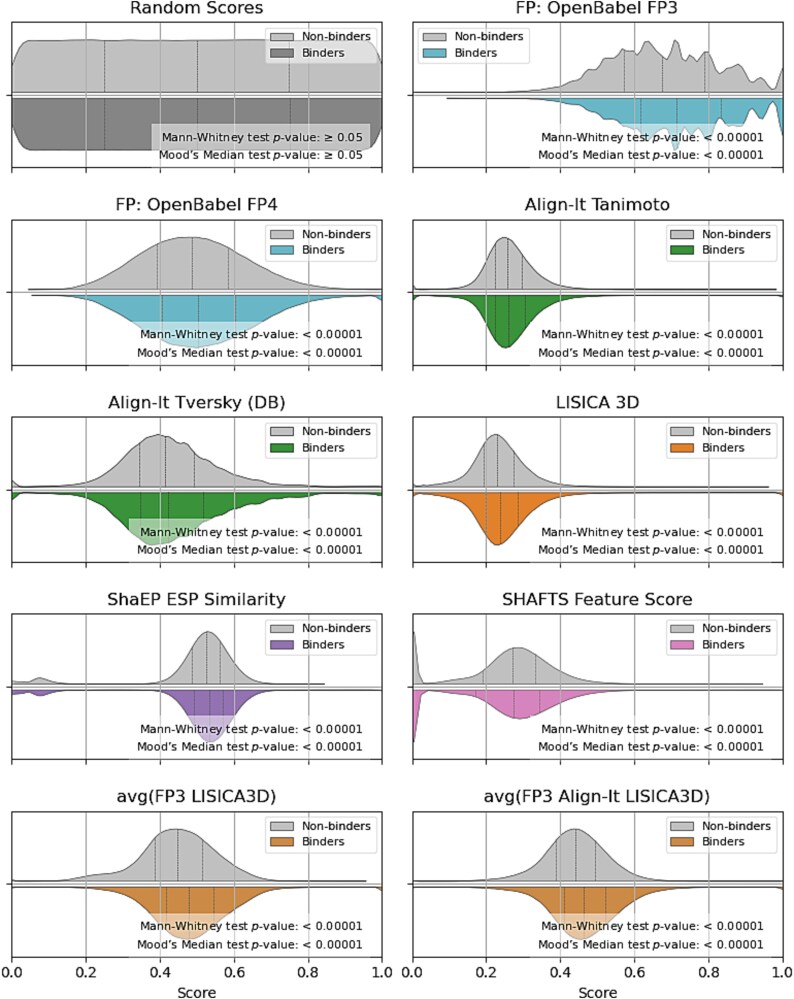
Violin plots, drawn using a kernel density estimate (KDE), showing the distribution of similarity scores for selected methods, split by the binding class (*non-binders* and *binders*; for the *non-binders*, the samples were randomly drawn in a number equal to the number of *binders*). Random scores serve as a negative control. Quartile lines indicate medians and interquartile ranges. *p*-values were calculated using the Mann–Whitney U test (compares the central tendencies of *binders* and *non-binders* score distributions) and Mood’s median test (evaluates differences in medians).

In this analysis, we employed the Mann–Whitney U test and Mood’s median test to evaluate differences in similarity scores between *binders* and *non-binders* across various methods (Fig. 5 and Tables S1–S5 in Supplementary materials). The Mann–Whitney U test, a non-parametric test, compares the rank distributions of two independent groups, providing insights into whether one group generally exhibits higher or lower values than the other. This test helps determine if the overall similarity score distributions for *binders* are significantly different from those of *non-binders* The Mood’s Median Test, another non-parametric method, specifically assesses whether the medians of the two groups differ, focusing on central tendency without being influenced by the shape or spread of the distributions. Among the evaluated methods, only the negative control (random scores) yielded non-significant *p*-values (Mann–Whitney *p*-value: .917; Mood Median Test *p*-value: .859), indicating no meaningful difference in similarity scores between groups. For all other methods, both tests revealed highly significant results (*p*-value <.00001), indicating that *binders* consistently exhibit higher similarity score distributions than *non-binders*. This finding reflects the methods’ general ability to discriminate between binding and non-binding compounds, even if the average classification performance (i.e. a grand average of all parameter settings without any method optimization) is only slightly above that of random classification. The agreement of these tests highlights not only differences in distribution but also a consistent shift in central tendency toward higher similarity scores for the *binders* group.

### Ligand-based versus structure-based methods

**Figure 6:**
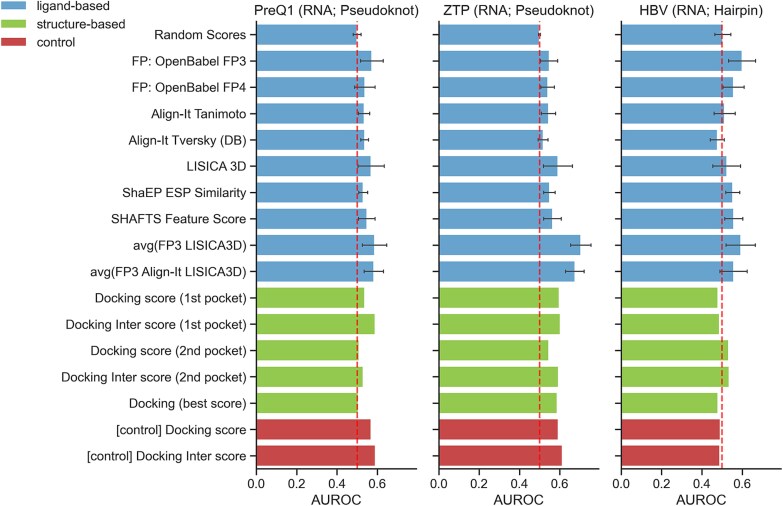
Comparison of the performance of selected ligand-based methods, structure-based molecular docking for three molecular targets. Docking to an unrelated protein target (JAK2 kinase) serves as a control. For ligand-based methods, the average values are shown, and error bars represent standard deviation. The dashed vertical line at AUC = 0.50 indicates the baseline for classification performance.

The primary challenge in structure-based screening methods is the availability of reliable 3D models of target structures. Among the targets investigated in this study, only one structure with the same sequence used for screening was available—an HBV NMR model solved in the absence of a bound ligand. To provide a more comprehensive comparison between ligand- and structure-based approaches, we constructed additional models of preQ1 and ZTP riboswitches using Protenix—an open-source PyTorch reproduction of the AlphaFold 3 method. Since none of the target structures contained bound ligands, binding pocket prediction was required, presenting an additional methodological challenge. We employed DRLiPS for pocket prediction and selected the two highest-scoring pockets for each target. Molecular docking was performed independently for each predicted pocket using two scoring functions (“docking score” and “docking inter score”), yielding four sets of scores per target. We also evaluated the performance of combined docking results by selecting the best score obtained for each molecular target across all pockets and scoring functions. As a negative control, we used docking scores from an unrelated protein structure (JAK2 kinase). Results are summarized in Fig. 6 and Supplementary Table S7.

Ligand-based methods demonstrated better performance (measured by the average AUROC) compared to structure-based docking for two targets: HBV (0.600 versus 0.534) and ZTP (0.705 versus 0.602). For PreQ1, the AUROC values were nearly identical between ligand-based and structure-based methods (0.586 versus 0.589, respectively). Notably, for two molecular targets, the negative control experiment (docking to an unrelated protein structure) yielded comparable or superior performance to docking against the actual target structure: PreQ1 (0.590 versus 0.589) and ZTP (0.612 versus 0.602). These results highlight the current limitations of structure-based approaches for nucleic acid targets and underscore the practical utility of ligand-based methods in the absence of high-quality structural data with validated binding sites.

### Case study

**Figure 7:**
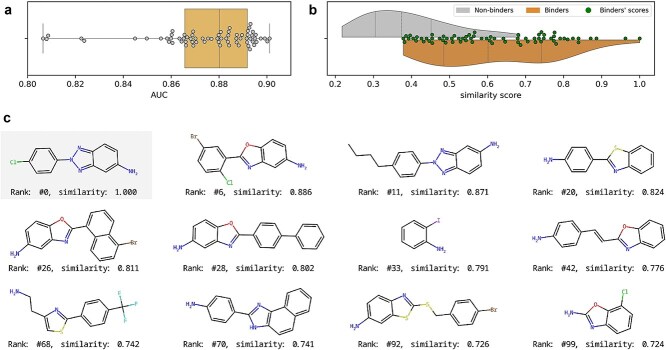
Analysis of the performance of the consensus method avg(FP3 Align-It LISICA3D) for glutamine riboswitch. (a) Distribution of AUC values over 70 template molecules. (b) Comparison of similarity scores to the template molecule, for *binders* and *non-binders*, with overlaid scores for *binders*. (c) Sample of true *binders* selected among top 100 ranked molecules; the overall ranking and the consensus similarity score (average) is shown below each molecule (the first molecule was used as the template).

Glutamine riboswitches are regulatory RNA elements that respond to the presence of L-glutamine, influencing gene expression related to nitrogen metabolism in response to fluctuating glutamine concentrations. We retrospectively analyzed the results of a Virtual Screening experiment using one of the best-performing consensus method—avg(FP3 Align-It LISICA3D), which combines the similarity scores of the OpenBabel FP3 fingerprint, Align-It Tversky similarity, and Lisica 3D similarity. For this RNA target, the average AUC of this method calculated for all templates was 0.875 ± 0.022 (Fig. 7, panel a), while BEDROC equaled 0.357 ± 0.106, and enrichment factor (1%) equaled 18.510 ± 12.974. The score distributions for *binders* and *non-binders* show a clear separation, with *binders* exhibiting consistently higher scores (panel b).

In the top 100 scored compounds (out of 7676), there were 22 true *binders* (other than the template molecule). In this case study, we focused mostly on the quality of the retrieved hits, i.e. if the method (or method combination) managed to retrieve hit compounds with different scaffolds than the one in the template molecule used for searching. Apart from molecules with the same as in the template benzotriazole scaffold, the method selected several other chemotypes as the most similar compounds. Apart from molecules having analogous heterocyclic fused rings, such as benzoxazole and benzothiazole, among top scored hits there are compounds with 2-phenylthiazole, aminobenzene, or 1H-naphth[1,2-d]imidazole scaffolds. In a real-life scenario, a particularly interesting finding of this screening campaign would be the discovery of binding fragments (molecules with a relatively small molecular mass), such as 2-iodoaniline (rank 33) or 7-chloro-1,3-benzoxazol-2-amine (rank 99), which could serve as starting points for the development of new—more potent—hits/leads based on established fragment growing or linking strategies. Among the top 10 hits we identified six unique Murcko scaffolds (and four unique Murcko frameworks), while among the top 30 hits there were 15 unique Murcko scaffolds (and 11 frameworks). In this setup, the consensus method achieved a very good performance: the AUC value was 0.895, the BEDROC value was 0.495 and the enrichment factor (1%) was 41.38. Such a variety of chemotypes selected during the screening would definitely allow medicinal chemists to elucidate the binding mechanism of the ligand to the glutamine riboswitch and enrich the structure–activity relationship landscape for this RNA target. Interestingly, to best of our knowledge, no synthetic small molecules beyond amino acid analogs have been reported to bind glutamine riboswitches.

In Supplementary Note 2, we present the analysis of VS for two additional nucleic acid targets, for which the performance was relatively good (MYC DNA) and moderate (MTOR DNA). In both cases, the consensus method, avg(FP3 Align-It LISICA3D), yielded fewer hits among the top 100 scored molecules compared to glutamine riboswitch. However, these hits belong to different chemotypes than the template compound. This confirms that the method extends beyond a simple framework or molecular similarity, focusing instead on more subtle structural features that may be relevant for nucleic acid binding.

## Summary

Computational methods play a central role in the early stages of drug discovery and are widely used in virtual screening. With the increasing recognition of the roles of RNA and DNA in disease, there is growing interest in using nucleic acids as potential targets for novel therapies. Given the limited availability of experimentally determined RNA 3D structures and the still insufficient accuracy of *in silico* RNA structure prediction methods (compared to e.g. AlphaFold 3 for proteins [8]), there is a clear need for ligand-based methods that do not require target structure information. In this work, we have benchmarked a number of such algorithms, which were primarily designed for molecules targeting proteins, but by their nature can also be applied to RNA. We have shown that some ligand-based methods can be effectively used to build predictive models for RNA targets, while others yield performance close to random selection. We have uncovered a few unexpected trends in fingerprint selection regarding parameters such as fingerprint radius and demonstrated the benefits of consensus approaches with a small number of carefully selected, orthogonal screening methods. High-performance method combinations can be identified even when the average performance of individual methods is close to or below random, as shown in the case study of the glutamine riboswitch. We also demonstrated that for selected targets, ligand-based methods are more straightforward to implement and achieve better predictive performance.

This study provides valuable guidance to researchers wishing to apply computational methods to RNA-targeted drug discovery. It has the potential to accelerate the development of RNA-based therapeutics despite the limitations of structural data.

**Key Points**

- We evaluated ligand-based methods for selection of nucleic acid binding ligands.
- Simple substructure-based fingerprints (OpenBabel FP3) significantly outperform complex circular fingerprints for nucleic acid targets.
- We proposed a consensus method combining multiple scores—this new approach achieves superior performance.
- Performance varies across nucleic acid targets, highlighting the need for target-specific method selection in ribonucleic acid/deoxyribonucleic acid-targeted virtual screening campaigns.

## Supplementary Material

RNA_ligandbased_SI_bbaf620

## Data Availability

The parts of the dataset, the processed data, descriptors, and the code used for analyses can be found at https://github.com/filipsPL/rna-ligand-based (https://doi.org/10.5281/zenodo.15683151). Given the large size of the complete raw dataset, full detailed virtual screening results are available upon request from the corresponding author.
