# Supplementary material for: Evaluation of single-template ligand-based methods for the discovery of small-molecule nucleic acid binders
Source: Brief Bioinform. 2025 Nov 21;26(6):bbaf620. doi: 10.1093/bib/bbaf620 (PMC12636511; doi:10.1093/bib/bbaf620)
Supplement: RNA_ligandbased_SI_bbaf620 [file rna_ligandbased_si_bbaf620.docx]

**Supplementary material**

Evaluation of single-template ligand-based methods for the discovery of small-molecule nucleic acid binders.

Dávid Bajusz^1^, Anita Rácz^2^, Janusz M. Bujnicki ^3^, Filip Stefaniak ^3,*^

1 Medicinal Chemistry Research Group, HUN-REN Research Centre for Natural Sciences, Magyar tudósok krt. 2, 1117 Budapest, Hungary

2 Plasma Chemistry Research Group, HUN-REN Research Centre for Natural Sciences, Magyar tudósok krt. 2, 1117 Budapest, Hungary

3 Laboratory of Bioinformatics and Protein Engineering, International Institute of Molecular and Cell Biology in Warsaw, 4 Ks. Trojdena Str., 02-109 Warsaw, Poland

^*^ Corresponding author (Laboratory of Bioinformatics and Protein Engineering, International Institute of Molecular and Cell Biology in Warsaw, ul. Ks. Trojdena 4, 02-109 Warsaw, Poland; tel: (+48-22) 597-07-53, fax: (+48-22) 597-07-15; [fstefaniak@iimcb.gov.pl](mailto:fstefaniak@iimcb.gov.pl)).


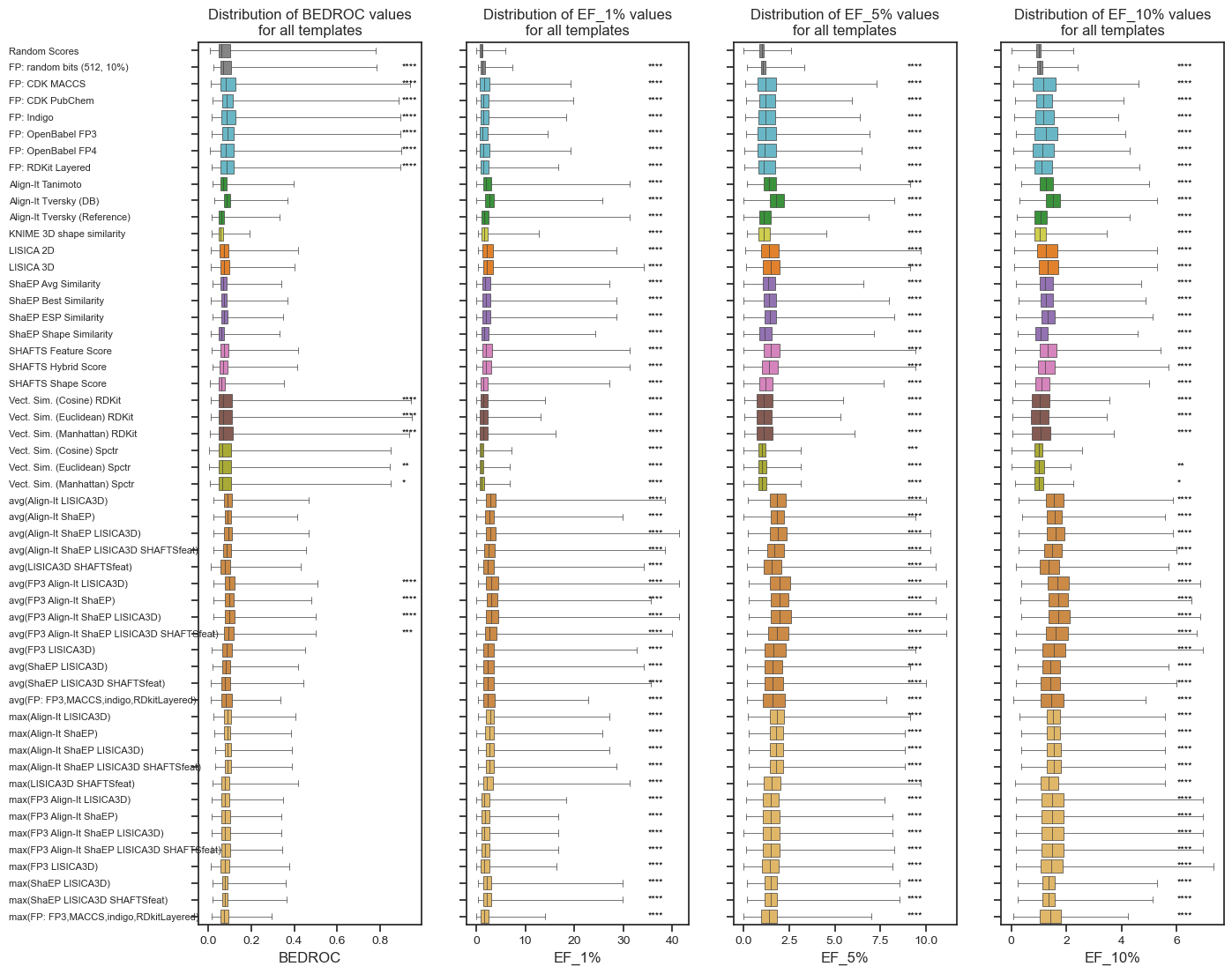
**Figure S1**. Comparison of the performance for ligand-based methods for all nucleic acid targets. The significance levels are shown next to each boxplot for methods for which the mean performance value was higher than for ‘Random Scores’ and are denoted by asterisks (* p < 0.05, ** p < 0.01, *** p < 0.001, **** p < 0.0001).


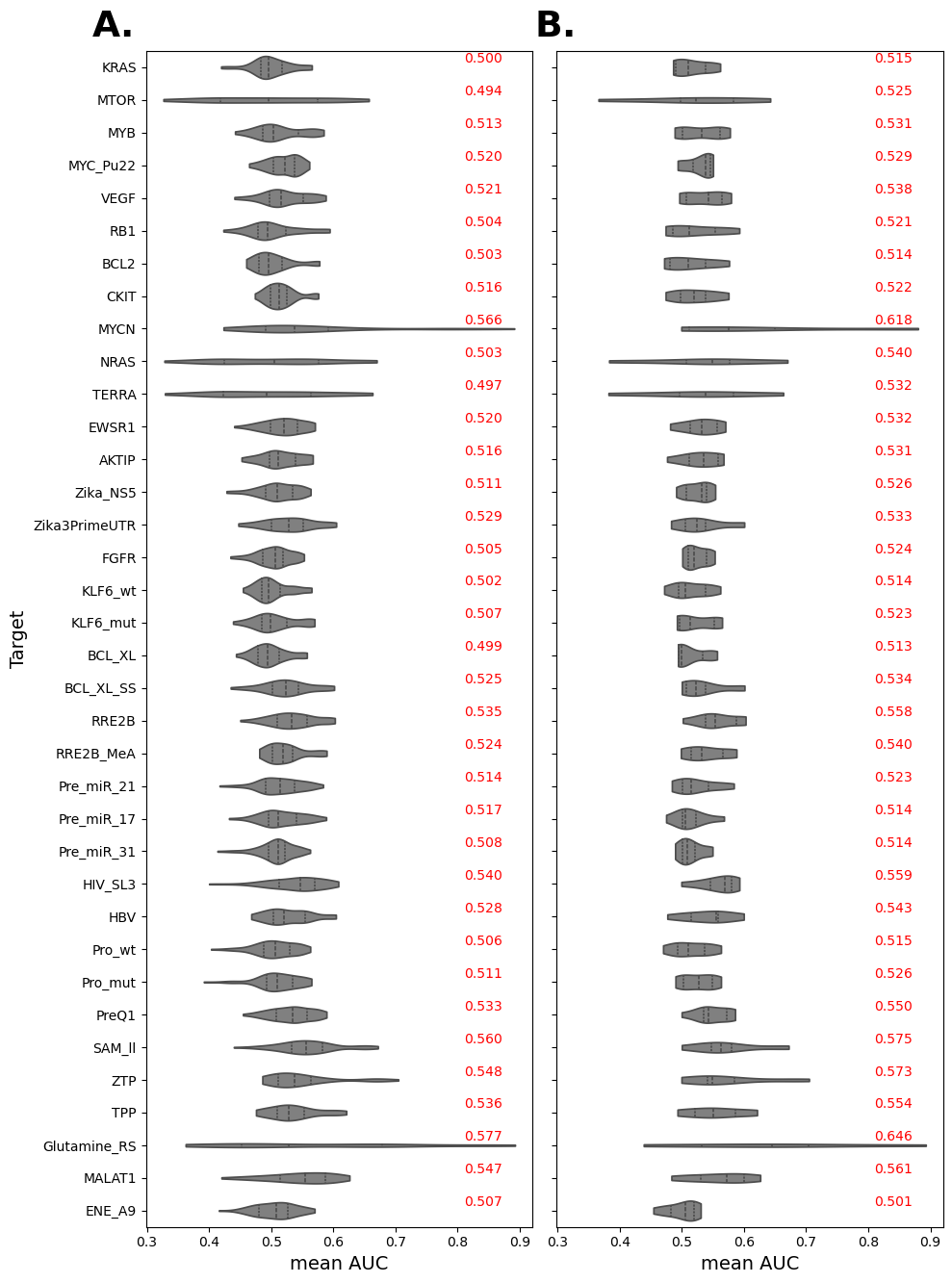


**Figure S2.** The distribution of the AUC values for individual molecular targets. **A.** For all methods, **B.** for selected best methods (as listed in Figure 4). Mean AUC values are shown in red.


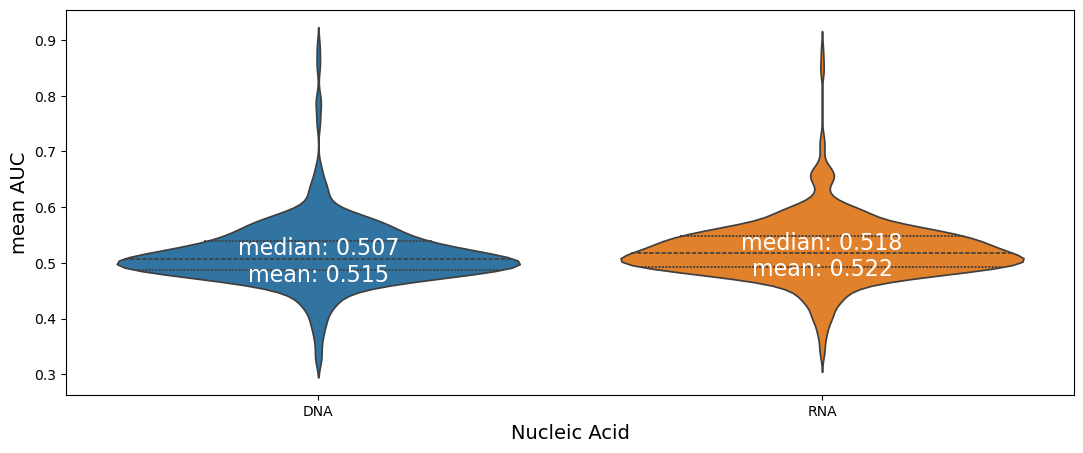
**Figure S3.** The distribution of the AUC values for all methods for DNA and RNA.


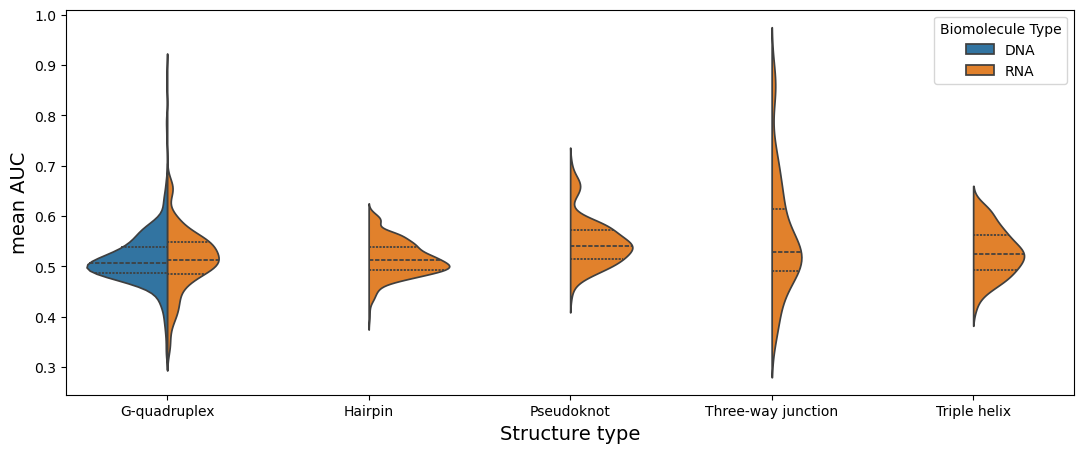
**Figure S4.** The distribution of the AUC values for all methods for various structure types.


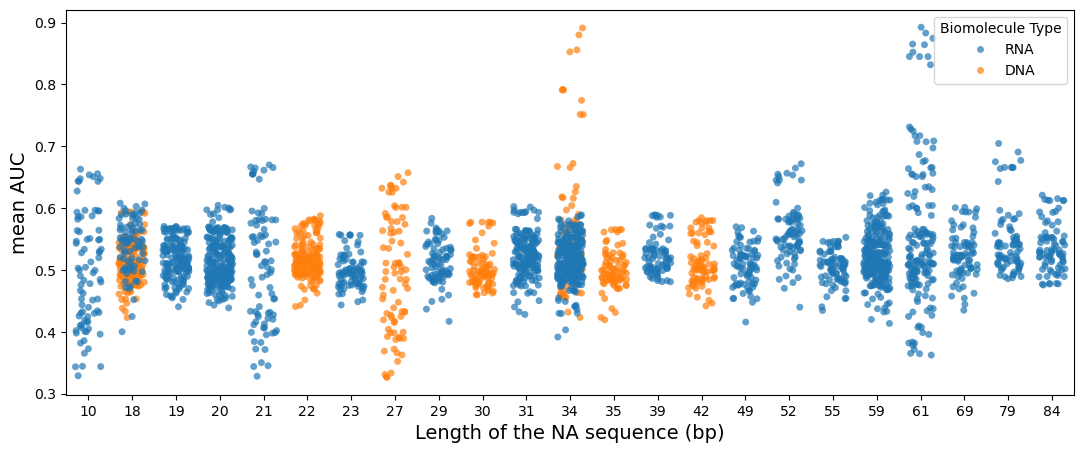
**Figure S5.** The AUC values for all methods for molecular targets of different lengths.

**Table S1.** Mean values and p-values for the AUROC

| **Method name** | **p-value** | **mean AUC** |
| --- | --- | --- |
| Align-It Tanimoto | 3.216E-132 | 0.51686 |
| Align-It Tversky (DB) | 0.000E+00 | 0.53069 |
| Align-It Tversky (Reference) | 4.090E-17 | 0.49431 |
| FP: CDK ECFP0 | 6.630E-16 | 0.50931 |
| FP: CDK ECFP2 | 2.068E-11 | 0.50751 |
| FP: CDK ECFP4 | 4.936E-03 | 0.50282 |
| FP: CDK Estate | 6.117E-13 | 0.50825 |
| FP: CDK FCFP0 | 1.243E-72 | 0.47883 |
| FP: CDK FCFP2 | 2.179E-02 | 0.49737 |
| FP: CDK FCFP4 | 3.229E-02 | 0.49761 |
| FP: CDK MACCS | 2.750E-43 | 0.51865 |
| FP: CDK PubChem | 5.491E-59 | 0.51439 |
| FP: CDK Standard | 1.296E-12 | 0.50780 |
| FP: Indigo | 2.285E-95 | 0.52161 |
| FP: MAP4 (1024 bits) | 1.058E-43 | 0.48853 |
| FP: MAP4 (2048 bits) | 9.322E-16 | 0.49288 |
| FP: MAP4 (256 bits) | 1.979E-121 | 0.47901 |
| FP: MAP4 (512 bits) | 1.739E-81 | 0.48379 |
| FP: OpenBabel FP2 | 6.508E-46 | 0.51427 |
| FP: OpenBabel FP3 | 0.000E+00 | 0.55092 |
| FP: OpenBabel FP4 | 2.550E-77 | 0.52203 |
| FP: RDKit | 1.101E-169 | 0.47009 |
| FP: RDKit AtomPair | 6.129E-02 | 0.49748 |
| FP: RDKit Avalon | 8.865E-05 | 0.50426 |
| FP: RDKit FeatAtom Morgan | 5.402E-16 | 0.50927 |
| FP: RDKit Layered | 1.184E-33 | 0.51328 |
| FP: RDKit Morgan | 9.935E-02 | 0.50164 |
| FP: RDKit Torsion | 1.871E-26 | 0.51093 |
| FP: random bits (512, 10%) | 6.555E-10 | 0.50258 |
| KNIME 3D shape similarity | 1.327E-47 | 0.49068 |
| LISICA 2D | 1.455E-170 | 0.52862 |
| LISICA 3D | 1.285E-263 | 0.53588 |
| SHAFTS Feature Score | 1.377E-62 | 0.51443 |
| SHAFTS Hybrid Score | 4.743E-14 | 0.50668 |
| SHAFTS Shape Score | 7.746E-06 | 0.49610 |
| ShaEP Avg Similarity | 2.242E-192 | 0.52093 |
| ShaEP Best Similarity | 2.568E-170 | 0.51987 |
| ShaEP ESP Similarity | 0.000E+00 | 0.53581 |
| ShaEP Shape Similarity | 8.899E-01 | 0.49981 |
| Vect. Sim. (Cosine) RDKit | 4.001E-02 | 0.50266 |
| Vect. Sim. (Cosine) Spctr | 2.553E-10 | 0.49651 |
| Vect. Sim. (Euclidean) RDKit | 3.485E-01 | 0.50111 |
| Vect. Sim. (Euclidean) Spctr | 3.033E-09 | 0.49611 |
| Vect. Sim. (Manhattan) RDKit | 4.136E-03 | 0.50374 |
| Vect. Sim. (Manhattan) Spctr | 1.861E-14 | 0.49503 |
| avg(Align-It LISICA3D) | 0.000E+00 | 0.54125 |
| avg(Align-It ShaEP LISICA3D SHAFTSfeat) | 0.000E+00 | 0.53550 |
| avg(Align-It ShaEP LISICA3D) | 0.000E+00 | 0.54640 |
| avg(Align-It ShaEP) | 0.000E+00 | 0.53992 |
| avg(FP3 Align-It LISICA3D) | 0.000E+00 | 0.59121 |
| avg(FP3 Align-It ShaEP LISICA3D SHAFTSfeat) | 0.000E+00 | 0.57369 |
| avg(FP3 Align-It ShaEP LISICA3D) | 0.000E+00 | 0.58548 |
| avg(FP3 Align-It ShaEP) | 0.000E+00 | 0.58285 |
| avg(FP3 LISICA3D) | 0.000E+00 | 0.59513 |
| avg(FP: FP3,MACCS,indigo,RDkitLayered) | 0.000E+00 | 0.55344 |
| avg(LISICA3D SHAFTSfeat) | 1.131E-109 | 0.52168 |
| avg(ShaEP LISICA3D SHAFTSfeat) | 5.737E-160 | 0.52590 |
| avg(ShaEP LISICA3D) | 0.000E+00 | 0.54624 |
| max(Align-It LISICA3D) | 0.000E+00 | 0.53226 |
| max(Align-It ShaEP LISICA3D SHAFTSfeat) | 0.000E+00 | 0.54923 |
| max(Align-It ShaEP LISICA3D) | 0.000E+00 | 0.54883 |
| max(Align-It ShaEP) | 0.000E+00 | 0.54859 |
| max(FP3 Align-It LISICA3D) | 0.000E+00 | 0.59428 |
| max(FP3 Align-It ShaEP LISICA3D SHAFTSfeat) | 0.000E+00 | 0.59137 |
| max(FP3 Align-It ShaEP LISICA3D) | 0.000E+00 | 0.59134 |
| max(FP3 Align-It ShaEP) | 0.000E+00 | 0.59133 |
| max(FP3 LISICA3D) | 0.000E+00 | 0.59508 |
| max(FP: FP3,MACCS,indigo,RDkitLayered) | 0.000E+00 | 0.56289 |
| max(LISICA3D SHAFTSfeat) | 2.192E-218 | 0.52984 |
| max(ShaEP LISICA3D SHAFTSfeat) | 0.000E+00 | 0.53803 |
| max(ShaEP LISICA3D) | 0.000E+00 | 0.53765 |

**Table S2.** Mean values and p-values for the BEDROC

| **Method name** | **p-value** | **mean BEDROC** |
| --- | --- | --- |
| Align-It Tanimoto | 1.1324E-42 | 0.076886 |
| Align-It Tversky (DB) | 6.2536E-05 | 0.091985 |
| Align-It Tversky (Reference) | 3.0748E-95 | 0.064071 |
| FP: CDK ECFP0 | 2.6380E-33 | 0.108686 |
| FP: CDK ECFP2 | 4.1867E-28 | 0.108672 |
| FP: CDK ECFP4 | 9.2353E-21 | 0.106381 |
| FP: CDK Estate | 7.2049E-40 | 0.109932 |
| FP: CDK FCFP0 | 7.7497E-07 | 0.102492 |
| FP: CDK FCFP2 | 3.3140E-30 | 0.107893 |
| FP: CDK FCFP4 | 6.3015E-26 | 0.107114 |
| FP: CDK MACCS | 1.9357E-53 | 0.113073 |
| FP: CDK PubChem | 1.7395E-94 | 0.113419 |
| FP: CDK Standard | 1.6965E-55 | 0.111067 |
| FP: Indigo | 1.3241E-86 | 0.114350 |
| FP: MAP4 (1024 bits) | 3.6650E-36 | 0.106749 |
| FP: MAP4 (2048 bits) | 1.6642E-30 | 0.107075 |
| FP: MAP4 (256 bits) | 1.1847E-25 | 0.106194 |
| FP: MAP4 (512 bits) | 1.8637E-34 | 0.107288 |
| FP: OpenBabel FP2 | 3.9803E-60 | 0.111560 |
| FP: OpenBabel FP3 | 1.8763E-109 | 0.116649 |
| FP: OpenBabel FP4 | 6.2105E-59 | 0.113138 |
| FP: RDKit | 3.1323E-01 | 0.099095 |
| FP: RDKit AtomPair | 9.3255E-24 | 0.107420 |
| FP: RDKit Avalon | 1.3379E-34 | 0.108103 |
| FP: RDKit FeatAtom Morgan | 4.4987E-31 | 0.109387 |
| FP: RDKit Layered | 2.2562E-68 | 0.111921 |
| FP: RDKit Morgan | 2.2227E-19 | 0.106042 |
| FP: RDKit Torsion | 2.3354E-50 | 0.110314 |
| FP: random bits (512, 10%) | 9.2033E-83 | 0.103231 |
| KNIME 3D shape similarity | 4.5366E-107 | 0.061612 |
| LISICA 2D | 3.3014E-25 | 0.081713 |
| LISICA 3D | 2.7852E-21 | 0.083727 |
| SHAFTS Feature Score | 1.1404E-26 | 0.081910 |
| SHAFTS Hybrid Score | 2.3929E-39 | 0.078264 |
| SHAFTS Shape Score | 3.4885E-88 | 0.066974 |
| ShaEP Avg Similarity | 5.6049E-51 | 0.074950 |
| ShaEP Best Similarity | 8.2196E-45 | 0.076936 |
| ShaEP ESP Similarity | 1.9600E-37 | 0.078103 |
| ShaEP Shape Similarity | 2.7751E-92 | 0.066062 |
| Vect. Sim. (Cosine) RDKit | 7.7078E-20 | 0.106440 |
| Vect. Sim. (Cosine) Spctr | 1.9715E-01 | 0.098804 |
| Vect. Sim. (Euclidean) RDKit | 3.0079E-15 | 0.105273 |
| Vect. Sim. (Euclidean) Spctr | 3.8227E-04 | 0.099723 |
| Vect. Sim. (Manhattan) RDKit | 1.5443E-16 | 0.106089 |
| Vect. Sim. (Manhattan) Spctr | 4.2300E-03 | 0.099466 |
| avg(Align-It LISICA3D) | 6.7269E-01 | 0.097618 |
| avg(Align-It ShaEP LISICA3D SHAFTSfeat) | 9.4651E-04 | 0.093272 |
| avg(Align-It ShaEP LISICA3D) | 5.6207E-01 | 0.099128 |
| avg(Align-It ShaEP) | 5.5457E-03 | 0.094015 |
| avg(FP3 Align-It LISICA3D) | 4.6650E-18 | 0.109721 |
| avg(FP3 Align-It ShaEP LISICA3D SHAFTSfeat) | 1.4481E-05 | 0.104055 |
| avg(FP3 Align-It ShaEP LISICA3D) | 8.9617E-20 | 0.110331 |
| avg(FP3 Align-It ShaEP) | 4.1263E-10 | 0.106897 |
| avg(FP3 LISICA3D) | 8.0671E-01 | 0.097935 |
| avg(FP: FP3,MACCS,indigo,RDkitLayered) | 4.3494E-04 | 0.092281 |
| avg(LISICA3D SHAFTSfeat) | 4.0768E-16 | 0.085846 |
| avg(ShaEP LISICA3D SHAFTSfeat) | 2.4704E-10 | 0.088678 |
| avg(ShaEP LISICA3D) | 7.1832E-10 | 0.088992 |
| max(Align-It LISICA3D) | 1.5103E-03 | 0.093315 |
| max(Align-It ShaEP LISICA3D SHAFTSfeat) | 2.8652E-03 | 0.093595 |
| max(Align-It ShaEP LISICA3D) | 1.7970E-03 | 0.093366 |
| max(Align-It ShaEP) | 1.7620E-04 | 0.092367 |
| max(FP3 Align-It LISICA3D) | 5.0064E-14 | 0.087738 |
| max(FP3 Align-It ShaEP LISICA3D SHAFTSfeat) | 9.4949E-14 | 0.087831 |
| max(FP3 Align-It ShaEP LISICA3D) | 1.0416E-13 | 0.087837 |
| max(FP3 Align-It ShaEP) | 1.3137E-13 | 0.087879 |
| max(FP3 LISICA3D) | 2.0759E-17 | 0.086569 |
| max(FP: FP3,MACCS,indigo,RDkitLayered) | 2.9747E-26 | 0.079877 |
| max(LISICA3D SHAFTSfeat) | 5.9177E-18 | 0.085153 |
| max(ShaEP LISICA3D SHAFTSfeat) | 5.5009E-30 | 0.080525 |
| max(ShaEP LISICA3D) | 4.4949E-32 | 0.079792 |

**Table S3.** Mean values and p-values for the Enrichment Factor EF(1%)

| **Method name** | **p-value** | **mean EF_1%** |
| --- | --- | --- |
| Align-It Tanimoto | 0.000E+00 | 2.537202 |
| Align-It Tversky (DB) | 0.000E+00 | 2.867940 |
| Align-It Tversky (Reference) | 5.416E-213 | 2.048690 |
| FP: CDK ECFP0 | 7.161E-188 | 1.968105 |
| FP: CDK ECFP2 | 1.847E-180 | 2.165490 |
| FP: CDK ECFP4 | 1.867E-179 | 2.072511 |
| FP: CDK Estate | 1.414E-173 | 2.028258 |
| FP: CDK FCFP0 | 1.233E-72 | 1.395440 |
| FP: CDK FCFP2 | 2.754E-179 | 2.109195 |
| FP: CDK FCFP4 | 7.414E-182 | 2.103968 |
| FP: CDK MACCS | 1.306E-205 | 2.133953 |
| FP: CDK PubChem | 1.249E-193 | 2.094470 |
| FP: CDK Standard | 1.126E-180 | 2.056614 |
| FP: Indigo | 4.918E-202 | 2.084187 |
| FP: MAP4 (1024 bits) | 3.034E-164 | 1.887120 |
| FP: MAP4 (2048 bits) | 8.625E-176 | 2.035654 |
| FP: MAP4 (256 bits) | 3.206E-125 | 1.536710 |
| FP: MAP4 (512 bits) | 8.102E-152 | 1.740415 |
| FP: OpenBabel FP2 | 4.316E-185 | 2.072617 |
| FP: OpenBabel FP3 | 2.424E-125 | 1.641778 |
| FP: OpenBabel FP4 | 2.075E-196 | 2.145779 |
| FP: RDKit | 1.062E-167 | 1.882750 |
| FP: RDKit AtomPair | 1.381E-189 | 1.997209 |
| FP: RDKit Avalon | 3.191E-175 | 2.014482 |
| FP: RDKit FeatAtom Morgan | 1.664E-192 | 2.205371 |
| FP: RDKit Layered | 3.196E-210 | 2.039885 |
| FP: RDKit Morgan | 6.825E-181 | 2.062402 |
| FP: RDKit Torsion | 1.465E-194 | 2.079895 |
| FP: random bits (512, 10%) | 3.744E-169 | 1.456438 |
| KNIME 3D shape similarity | 0.000E+00 | 1.891175 |
| LISICA 2D | 0.000E+00 | 2.880538 |
| LISICA 3D | 0.000E+00 | 2.791078 |
| SHAFTS Feature Score | 0.000E+00 | 2.597072 |
| SHAFTS Hybrid Score | 4.751E-283 | 2.486045 |
| SHAFTS Shape Score | 1.898E-159 | 1.887127 |
| ShaEP Avg Similarity | 7.609E-298 | 2.289899 |
| ShaEP Best Similarity | 0.000E+00 | 2.399534 |
| ShaEP ESP Similarity | 0.000E+00 | 2.320829 |
| ShaEP Shape Similarity | 4.736E-239 | 1.964452 |
| Vect. Sim. (Cosine) RDKit | 1.140E-200 | 1.815366 |
| Vect. Sim. (Cosine) Spctr | 1.161E-13 | 1.119865 |
| Vect. Sim. (Euclidean) RDKit | 3.521E-194 | 1.804239 |
| Vect. Sim. (Euclidean) Spctr | 4.694E-21 | 1.153801 |
| Vect. Sim. (Manhattan) RDKit | 1.031E-176 | 1.887948 |
| Vect. Sim. (Manhattan) Spctr | 3.528E-21 | 1.155958 |
| avg(Align-It LISICA3D) | 0.000E+00 | 3.318240 |
| avg(Align-It ShaEP LISICA3D SHAFTSfeat) | 0.000E+00 | 3.152095 |
| avg(Align-It ShaEP LISICA3D) | 0.000E+00 | 3.332201 |
| avg(Align-It ShaEP) | 0.000E+00 | 2.962138 |
| avg(FP3 Align-It LISICA3D) | 0.000E+00 | 3.819007 |
| avg(FP3 Align-It ShaEP LISICA3D SHAFTSfeat) | 0.000E+00 | 3.503996 |
| avg(FP3 Align-It ShaEP LISICA3D) | 0.000E+00 | 3.820437 |
| avg(FP3 Align-It ShaEP) | 0.000E+00 | 3.593716 |
| avg(FP3 LISICA3D) | 0.000E+00 | 3.142530 |
| avg(FP: FP3,MACCS,indigo,RDkitLayered) | 0.000E+00 | 3.097982 |
| avg(LISICA3D SHAFTSfeat) | 0.000E+00 | 2.924773 |
| avg(ShaEP LISICA3D SHAFTSfeat) | 0.000E+00 | 2.987674 |
| avg(ShaEP LISICA3D) | 0.000E+00 | 2.946012 |
| max(Align-It LISICA3D) | 0.000E+00 | 3.014642 |
| max(Align-It ShaEP LISICA3D SHAFTSfeat) | 0.000E+00 | 2.996007 |
| max(Align-It ShaEP LISICA3D) | 0.000E+00 | 2.987484 |
| max(Align-It ShaEP) | 0.000E+00 | 2.852024 |
| max(FP3 Align-It LISICA3D) | 2.179E-234 | 2.104980 |
| max(FP3 Align-It ShaEP LISICA3D SHAFTSfeat) | 1.072E-248 | 2.117869 |
| max(FP3 Align-It ShaEP LISICA3D) | 1.281E-246 | 2.110689 |
| max(FP3 Align-It ShaEP) | 8.935E-253 | 2.111156 |
| max(FP3 LISICA3D) | 4.077E-234 | 2.054540 |
| max(FP: FP3,MACCS,indigo,RDkitLayered) | 1.835E-232 | 1.862692 |
| max(LISICA3D SHAFTSfeat) | 0.000E+00 | 2.798030 |
| max(ShaEP LISICA3D SHAFTSfeat) | 0.000E+00 | 2.523946 |
| max(ShaEP LISICA3D) | 0.000E+00 | 2.478315 |

**Table S4.** Mean values and p-values for the Enrichment Factor EF(5%)

| **Method name** | **p-value** | **mean EF_5%** |
| --- | --- | --- |
| Align-It Tanimoto | 0.000E+00 | 1.505343 |
| Align-It Tversky (DB) | 0.000E+00 | 1.890094 |
| Align-It Tversky (Reference) | 2.756E-103 | 1.217593 |
| FP: CDK ECFP0 | 5.264E-104 | 1.302346 |
| FP: CDK ECFP2 | 4.938E-116 | 1.354959 |
| FP: CDK ECFP4 | 2.830E-97 | 1.295157 |
| FP: CDK Estate | 9.269E-104 | 1.331305 |
| FP: CDK FCFP0 | 4.944E-66 | 1.181752 |
| FP: CDK FCFP2 | 1.092E-106 | 1.304459 |
| FP: CDK FCFP4 | 1.514E-108 | 1.305069 |
| FP: CDK MACCS | 2.548E-148 | 1.378871 |
| FP: CDK PubChem | 1.005E-180 | 1.370112 |
| FP: CDK Standard | 4.288E-119 | 1.309757 |
| FP: Indigo | 1.006E-163 | 1.379453 |
| FP: MAP4 (1024 bits) | 1.907E-64 | 1.177646 |
| FP: MAP4 (2048 bits) | 2.210E-82 | 1.236576 |
| FP: MAP4 (256 bits) | 1.179E-21 | 1.086063 |
| FP: MAP4 (512 bits) | 9.819E-44 | 1.133075 |
| FP: OpenBabel FP2 | 5.259E-135 | 1.340599 |
| FP: OpenBabel FP3 | 7.611E-145 | 1.369690 |
| FP: OpenBabel FP4 | 1.074E-139 | 1.380575 |
| FP: RDKit | 5.589E-22 | 1.111788 |
| FP: RDKit AtomPair | 3.133E-100 | 1.282645 |
| FP: RDKit Avalon | 1.013E-110 | 1.277695 |
| FP: RDKit FeatAtom Morgan | 3.752E-133 | 1.372424 |
| FP: RDKit Layered | 7.135E-139 | 1.341343 |
| FP: RDKit Morgan | 3.928E-95 | 1.287191 |
| FP: RDKit Torsion | 3.122E-140 | 1.352059 |
| FP: random bits (512, 10%) | 3.229E-48 | 1.087871 |
| KNIME 3D shape similarity | 1.138E-76 | 1.157660 |
| LISICA 2D | 1.199E-299 | 1.589495 |
| LISICA 3D | 0.000E+00 | 1.641474 |
| SHAFTS Feature Score | 0.000E+00 | 1.633477 |
| SHAFTS Hybrid Score | 1.267E-299 | 1.553865 |
| SHAFTS Shape Score | 1.940E-133 | 1.298701 |
| ShaEP Avg Similarity | 0.000E+00 | 1.448188 |
| ShaEP Best Similarity | 0.000E+00 | 1.504765 |
| ShaEP ESP Similarity | 0.000E+00 | 1.519344 |
| ShaEP Shape Similarity | 2.819E-141 | 1.269421 |
| Vect. Sim. (Cosine) RDKit | 4.072E-76 | 1.214152 |
| Vect. Sim. (Cosine) Spctr | 1.393E-05 | 1.024696 |
| Vect. Sim. (Euclidean) RDKit | 5.245E-72 | 1.205484 |
| Vect. Sim. (Euclidean) Spctr | 2.636E-09 | 1.036485 |
| Vect. Sim. (Manhattan) RDKit | 1.512E-75 | 1.229791 |
| Vect. Sim. (Manhattan) Spctr | 2.355E-08 | 1.034165 |
| avg(Align-It LISICA3D) | 0.000E+00 | 1.983246 |
| avg(Align-It ShaEP LISICA3D SHAFTSfeat) | 0.000E+00 | 1.874813 |
| avg(Align-It ShaEP LISICA3D) | 0.000E+00 | 2.014547 |
| avg(Align-It ShaEP) | 0.000E+00 | 1.925322 |
| avg(FP3 Align-It LISICA3D) | 0.000E+00 | 2.207882 |
| avg(FP3 Align-It ShaEP LISICA3D SHAFTSfeat) | 0.000E+00 | 2.090949 |
| avg(FP3 Align-It ShaEP LISICA3D) | 0.000E+00 | 2.225962 |
| avg(FP3 Align-It ShaEP) | 0.000E+00 | 2.153770 |
| avg(FP3 LISICA3D) | 0.000E+00 | 1.919325 |
| avg(FP: FP3,MACCS,indigo,RDkitLayered) | 0.000E+00 | 1.809035 |
| avg(LISICA3D SHAFTSfeat) | 0.000E+00 | 1.707243 |
| avg(ShaEP LISICA3D SHAFTSfeat) | 0.000E+00 | 1.765481 |
| avg(ShaEP LISICA3D) | 0.000E+00 | 1.748781 |
| max(Align-It LISICA3D) | 0.000E+00 | 1.909699 |
| max(Align-It ShaEP LISICA3D SHAFTSfeat) | 0.000E+00 | 1.894153 |
| max(Align-It ShaEP LISICA3D) | 0.000E+00 | 1.888037 |
| max(Align-It ShaEP) | 0.000E+00 | 1.875070 |
| max(FP3 Align-It LISICA3D) | 0.000E+00 | 1.666010 |
| max(FP3 Align-It ShaEP LISICA3D SHAFTSfeat) | 0.000E+00 | 1.678389 |
| max(FP3 Align-It ShaEP LISICA3D) | 0.000E+00 | 1.674957 |
| max(FP3 Align-It ShaEP) | 0.000E+00 | 1.677073 |
| max(FP3 LISICA3D) | 2.188E-279 | 1.621308 |
| max(FP: FP3,MACCS,indigo,RDkitLayered) | 2.996E-256 | 1.507911 |
| max(LISICA3D SHAFTSfeat) | 0.000E+00 | 1.685186 |
| max(ShaEP LISICA3D SHAFTSfeat) | 0.000E+00 | 1.562812 |
| max(ShaEP LISICA3D) | 0.000E+00 | 1.546293 |

**Table S5.** Mean values and p-values for the Enrichment Factor EF(10%)

| **Method name** | **p-value** | **mean EF_10%** |
| --- | --- | --- |
| Align-It Tanimoto | 0.000E+00 | 1.307072 |
| Align-It Tversky (DB) | 0.000E+00 | 1.569531 |
| Align-It Tversky (Reference) | 8.208E-58 | 1.100325 |
| FP: CDK ECFP0 | 3.071E-76 | 1.178274 |
| FP: CDK ECFP2 | 2.103E-85 | 1.196317 |
| FP: CDK ECFP4 | 2.733E-67 | 1.159285 |
| FP: CDK Estate | 1.409E-69 | 1.171563 |
| FP: CDK FCFP0 | 4.493E-36 | 1.096229 |
| FP: CDK FCFP2 | 1.490E-72 | 1.162329 |
| FP: CDK FCFP4 | 6.740E-71 | 1.159933 |
| FP: CDK MACCS | 1.024E-123 | 1.237788 |
| FP: CDK PubChem | 3.155E-173 | 1.224742 |
| FP: CDK Standard | 1.629E-101 | 1.188266 |
| FP: Indigo | 1.232E-163 | 1.248000 |
| FP: MAP4 (1024 bits) | 4.798E-27 | 1.067954 |
| FP: MAP4 (2048 bits) | 1.296E-46 | 1.108463 |
| FP: MAP4 (256 bits) | 1.522E-02 | 1.010564 |
| FP: MAP4 (512 bits) | 2.293E-10 | 1.034451 |
| FP: OpenBabel FP2 | 7.209E-115 | 1.204990 |
| FP: OpenBabel FP3 | 2.108E-198 | 1.297078 |
| FP: OpenBabel FP4 | 1.192E-122 | 1.240204 |
| FP: RDKit | 1.581E-01 | 0.984201 |
| FP: RDKit AtomPair | 1.643E-64 | 1.155609 |
| FP: RDKit Avalon | 3.798E-81 | 1.153171 |
| FP: RDKit FeatAtom Morgan | 1.381E-102 | 1.210553 |
| FP: RDKit Layered | 3.588E-124 | 1.220132 |
| FP: RDKit Morgan | 5.332E-60 | 1.146908 |
| FP: RDKit Torsion | 9.310E-119 | 1.212912 |
| FP: random bits (512, 10%) | 1.477E-33 | 1.048632 |
| KNIME 3D shape similarity | 6.470E-37 | 1.073094 |
| LISICA 2D | 5.188E-282 | 1.370001 |
| LISICA 3D | 0.000E+00 | 1.414255 |
| SHAFTS Feature Score | 0.000E+00 | 1.401641 |
| SHAFTS Hybrid Score | 2.844E-268 | 1.335335 |
| SHAFTS Shape Score | 1.276E-110 | 1.178510 |
| ShaEP Avg Similarity | 0.000E+00 | 1.300878 |
| ShaEP Best Similarity | 0.000E+00 | 1.323127 |
| ShaEP ESP Similarity | 0.000E+00 | 1.359777 |
| ShaEP Shape Similarity | 3.160E-101 | 1.153650 |
| Vect. Sim. (Cosine) RDKit | 6.231E-40 | 1.112339 |
| Vect. Sim. (Cosine) Spctr | 2.737E-01 | 1.001082 |
| Vect. Sim. (Euclidean) RDKit | 4.272E-35 | 1.101706 |
| Vect. Sim. (Euclidean) Spctr | 7.768E-04 | 1.012621 |
| Vect. Sim. (Manhattan) RDKit | 1.454E-49 | 1.131818 |
| Vect. Sim. (Manhattan) Spctr | 1.383E-03 | 1.011668 |
| avg(Align-It LISICA3D) | 0.000E+00 | 1.638309 |
| avg(Align-It ShaEP LISICA3D SHAFTSfeat) | 0.000E+00 | 1.568731 |
| avg(Align-It ShaEP LISICA3D) | 0.000E+00 | 1.668922 |
| avg(Align-It ShaEP) | 0.000E+00 | 1.599082 |
| avg(FP3 Align-It LISICA3D) | 0.000E+00 | 1.837753 |
| avg(FP3 Align-It ShaEP LISICA3D SHAFTSfeat) | 0.000E+00 | 1.759453 |
| avg(FP3 Align-It ShaEP LISICA3D) | 0.000E+00 | 1.851839 |
| avg(FP3 Align-It ShaEP) | 0.000E+00 | 1.804160 |
| avg(FP3 LISICA3D) | 0.000E+00 | 1.680655 |
| avg(FP: FP3,MACCS,indigo,RDkitLayered) | 0.000E+00 | 1.549343 |
| avg(LISICA3D SHAFTSfeat) | 0.000E+00 | 1.442515 |
| avg(ShaEP LISICA3D SHAFTSfeat) | 0.000E+00 | 1.494528 |
| avg(ShaEP LISICA3D) | 0.000E+00 | 1.508902 |
| max(Align-It LISICA3D) | 0.000E+00 | 1.579181 |
| max(Align-It ShaEP LISICA3D SHAFTSfeat) | 0.000E+00 | 1.584046 |
| max(Align-It ShaEP LISICA3D) | 0.000E+00 | 1.580666 |
| max(Align-It ShaEP) | 0.000E+00 | 1.576407 |
| max(FP3 Align-It LISICA3D) | 0.000E+00 | 1.606313 |
| max(FP3 Align-It ShaEP LISICA3D SHAFTSfeat) | 0.000E+00 | 1.603172 |
| max(FP3 Align-It ShaEP LISICA3D) | 0.000E+00 | 1.603859 |
| max(FP3 Align-It ShaEP) | 0.000E+00 | 1.605120 |
| max(FP3 LISICA3D) | 0.000E+00 | 1.588064 |
| max(FP: FP3,MACCS,indigo,RDkitLayered) | 0.000E+00 | 1.446446 |
| max(LISICA3D SHAFTSfeat) | 0.000E+00 | 1.445963 |
| max(ShaEP LISICA3D SHAFTSfeat) | 0.000E+00 | 1.386048 |
| max(ShaEP LISICA3D) | 0.000E+00 | 1.378242 |

**Table S6.** Median values of AUC for fingerprints and similarity metrics.

| **Method** | **Braun-Blanquet** | **Cosine** | **Kulczynski** | **Russell-Raoy** | **Tanimoto** | **Tversky (Reference-weighted)** | **Tversky (Query-weighted)** | **Mean** |
| --- | --- | --- | --- | --- | --- | --- | --- | --- |
| FP: CDK ECFP0 | 0.492 | 0.498 | 0.499 | 0.475 | 0.497 | 0.490 | 0.506 | 0.495 |
| FP: CDK ECFP2 | 0.497 | 0.499 | 0.500 | 0.484 | 0.498 | 0.493 | 0.507 | 0.497 |
| FP: CDK ECFP4 | 0.490 | 0.495 | 0.496 | 0.485 | 0.494 | 0.490 | 0.499 | 0.493 |
| FP: CDK Estate | 0.493 | 0.495 | 0.496 | 0.471 | 0.495 | 0.486 | 0.505 | 0.493 |
| FP: CDK FCFP0 | 0.483 | 0.482 | 0.482 | 0.484 | 0.479 | 0.483 | 0.479 | 0.482 |
| FP: CDK FCFP2 | 0.487 | 0.492 | 0.493 | 0.481 | 0.490 | 0.486 | 0.496 | 0.490 |
| FP: CDK FCFP4 | 0.489 | 0.493 | 0.495 | 0.484 | 0.492 | 0.488 | 0.498 | 0.492 |
| FP: CDK MACCS | 0.506 | 0.506 | 0.507 | 0.476 | 0.506 | 0.492 | 0.522 | 0.503 |
| FP: CDK PubChem | 0.506 | 0.507 | 0.508 | 0.484 | 0.506 | 0.496 | 0.518 | 0.505 |
| FP: CDK Standard | 0.495 | 0.487 | 0.487 | 0.468 | 0.489 | 0.478 | 0.505 | 0.487 |
| FP: Indigo | 0.501 | 0.508 | 0.510 | 0.473 | 0.506 | 0.494 | 0.520 | 0.504 |
| FP: MAP4 (1024 bits) | 0.484 | 0.476 | 0.475 | 0.463 | 0.477 | 0.471 | 0.484 | 0.477 |
| FP: MAP4 (2048 bits) | 0.486 | 0.479 | 0.480 | 0.471 | 0.480 | 0.477 | 0.487 | 0.480 |
| FP: MAP4 (256 bits) | 0.478 | 0.457 | 0.455 | 0.450 | 0.459 | 0.454 | 0.469 | 0.460 |
| FP: MAP4 (512 bits) | 0.482 | 0.466 | 0.465 | 0.456 | 0.469 | 0.463 | 0.478 | 0.469 |
| FP: OpenBabel FP2 | 0.498 | 0.501 | 0.504 | 0.473 | 0.499 | 0.488 | 0.511 | 0.498 |
| FP: OpenBabel FP3 | 0.541 | 0.550 | 0.553 | 0.472 | 0.551 | 0.529 | 0.569 | 0.539 |
| FP: OpenBabel FP4 | 0.512 | 0.512 | 0.512 | 0.483 | 0.512 | 0.501 | 0.526 | 0.509 |
| FP: RDKit | 0.470 | 0.451 | 0.450 | 0.448 | 0.453 | 0.450 | 0.459 | 0.453 |
| FP: RDKit AtomPair | 0.481 | 0.483 | 0.486 | 0.470 | 0.482 | 0.475 | 0.493 | 0.482 |
| FP: RDKit Avalon | 0.490 | 0.484 | 0.486 | 0.465 | 0.486 | 0.476 | 0.503 | 0.484 |
| FP: RDKit FeatAtom Morgan | 0.504 | 0.508 | 0.509 | 0.496 | 0.506 | 0.502 | 0.512 | 0.506 |
| FP: RDKit Layered | 0.496 | 0.488 | 0.488 | 0.442 | 0.490 | 0.468 | 0.515 | 0.485 |
| FP: RDKit Morgan | 0.490 | 0.493 | 0.494 | 0.483 | 0.492 | 0.488 | 0.497 | 0.491 |
| FP: RDKit Torsion | 0.500 | 0.503 | 0.503 | 0.489 | 0.502 | 0.497 | 0.508 | 0.501 |
| FP: random bits (512, 10%) | 0.501 | 0.501 | 0.501 | 0.501 | 0.501 | 0.501 | 0.501 | 0.501 |
| Random Scores | 0.500 | 0.500 | 0.500 | 0.500 | 0.500 | 0.500 | 0.500 | 0.500 |
| **Mean** | 0.498 | 0.497 | 0.498 | 0.488 | 0.497 | 0.494 | 0.501 | 0.497 |

**Supplementary Note 1**

To identify chemical features that help to distinguish between binding and non-binding compounds in FP3 fingerprint, we performed a comprehensive statistical analysis of FP3 fingerprints across all molecular targets in our dataset. It covered 812799 small molecules, of which 4570 (0.6%) were classified as RNA binders. The analysis revealed 27 bits of FP3 fingerprint (49.1%) with statistically significant differences (p < 0.05), with the most discriminative features being aldehydes/ketones (bit2), hydroxy compounds (bit26), and alcohols (bit27), all showing negative associations with NA binding, while oximes (bit12) and anilines (bit53) demonstrated positive associations.


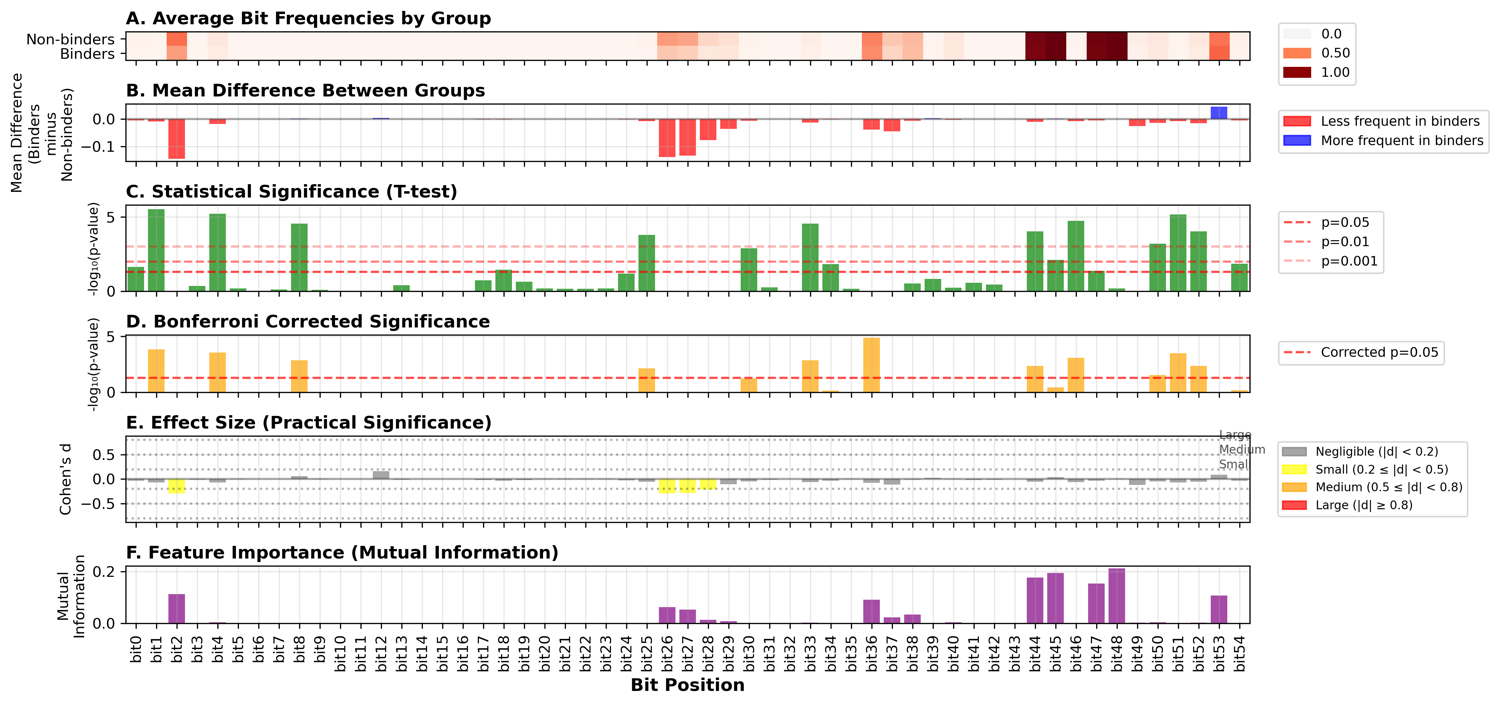
 **A.** The average frequency of each FP3 bit for binders and non-binders. as a heat map, with darker colors indicating higher frequencies. **B.** Mean difference in bit frequencies between groups, where red bars indicate features less frequent in binders and blue bars show features more frequent in binders. **C.** Statistical significance assessed using t-tests, with p-values displayed on a negative log10 scale, where higher bars indicate greater significance. **D.** Bonferroni-corrected p-values accounting for multiple testing across all 55 bits. **E.** Cohen's d effect sizes, with color coding indicating magnitude: gray (negligible), yellow (small), orange (medium), and red (large effects). **F.** Mutual information scores quantifying the information content of each bit for distinguishing between binders and non-binders.

Top 30 bits of FP3 fingerprint with most significant difference bewteen binders and non-binders:

| **FP3**  **bit** | **Non-binders Mean** | **Binders Mean** | **Mean Difference (Binders - Non-binders)** | **T-test P-value** | **Cohen's d (Effect Size)** | **Effect Size Interpretation** | **Bonferroni Corrected P-value (T-test)** | **Bonferroni Corrected P-value (Mann-Whitney)** | **Bonferroni Corrected P-value (Chi-square)** |
| --- | --- | --- | --- | --- | --- | --- | --- | --- | --- |
| bit2 | 0.486595 | 0.341575 | -0.145019 | 0.00E+00 | -0.29022 | small | 0.00E+00 | 0.00E+00 | 0.00E+00 |
| bit12 | 0.000562 | 0.004376 | 0.003815 | 0.00E+00 | 0.15802 | negligible | 0.00E+00 | 0.00E+00 | 0.00E+00 |
| bit26 | 0.348662 | 0.210066 | -0.138597 | 0.00E+00 | -0.29106 | small | 0.00E+00 | 0.00E+00 | 0.00E+00 |
| bit27 | 0.319883 | 0.186652 | -0.133231 | 0.00E+00 | -0.28588 | small | 0.00E+00 | 0.00E+00 | 0.00E+00 |
| bit28 | 0.15624 | 0.079869 | -0.076372 | 0.00E+00 | -0.21060 | small | 0.00E+00 | 0.00E+00 | 0.00E+00 |
| bit29 | 0.122302 | 0.08709 | -0.035212 | 0.00E+00 | -0.10755 | negligible | 0.00E+00 | 0.00E+00 | 0.00E+00 |
| bit36 | 0.429242 | 0.391466 | -0.037776 | 0.00E+00 | -0.07633 | negligible | 1.30E-05 | 1.50E-05 | 1.40E-05 |
| bit37 | 0.208513 | 0.163676 | -0.044837 | 0.00E+00 | -0.11042 | negligible | 0.00E+00 | 0.00E+00 | 0.00E+00 |
| bit49 | 0.045539 | 0.020569 | -0.02497 | 0.00E+00 | -0.11995 | negligible | 0.00E+00 | 0.00E+00 | 0.00E+00 |
| bit53 | 0.472266 | 0.517943 | 0.045677 | 0.00E+00 | 0.09150 | negligible | 0.00E+00 | 0.00E+00 | 0.00E+00 |
| bit1 | 0.015251 | 0.006783 | -0.008467 | 3.00E-06 | -0.06920 | negligible | 1.45E-04 | 1.70E-04 | 1.95E-04 |
| bit4 | 0.078666 | 0.060613 | -0.018053 | 6.00E-06 | -0.06710 | negligible | 2.86E-04 | 3.35E-04 | 3.26E-04 |
| bit51 | 0.014248 | 0.006346 | -0.007903 | 7.00E-06 | -0.06679 | negligible | 3.16E-04 | 3.70E-04 | 4.24E-04 |
| bit46 | 0.016103 | 0.008096 | -0.008007 | 1.80E-05 | -0.06370 | negligible | 8.25E-04 | 9.65E-04 | 1.07E-03 |
| bit8 | 0.000202 | 0.001094 | 0.000892 | 2.90E-05 | 0.06208 | negligible | 1.34E-03 | 1.57E-03 | 1.14E-02 |
| bit33 | 0.043249 | 0.030635 | -0.012614 | 2.90E-05 | -0.06206 | negligible | 1.35E-03 | 1.58E-03 | 1.58E-03 |
| bit44 | 0.967411 | 0.957112 | -0.0103 | 9.30E-05 | -0.05796 | negligible | 4.39E-03 | 5.14E-03 | 5.21E-03 |
| bit52 | 0.075701 | 0.060394 | -0.015307 | 9.50E-05 | -0.05790 | negligible | 4.46E-03 | 5.22E-03 | 5.01E-03 |
| bit25 | 0.019023 | 0.011379 | -0.007645 | 1.59E-04 | -0.05602 | negligible | 7.47E-03 | 8.75E-03 | 9.28E-03 |
| bit50 | 0.082192 | 0.068271 | -0.013921 | 6.30E-04 | -0.05071 | negligible | 2.96E-02 | 3.47E-02 | 3.27E-02 |
| bit30 | 0.019506 | 0.01291 | -0.006595 | 1.29E-03 | -0.04774 | negligible | 6.07E-02 | 7.10E-02 | 7.31E-02 |
| bit45 | 0.997663 | 0.999562 | 0.0019 | 7.86E-03 | 0.03943 | negligible | 3.70E-01 | 4.32E-01 | 5.77E-01 |
| bit54 | 0.022725 | 0.017287 | -0.005438 | 1.38E-02 | -0.03652 | negligible | 6.50E-01 | 7.61E-01 | 7.46E-01 |
| bit34 | 0.001702 | 0.000219 | -0.001484 | 1.50E-02 | -0.03608 | negligible | 7.06E-01 | 8.26E-01 | 1.00E+00 |
| bit0 | 0.018783 | 0.014223 | -0.00456 | 2.35E-02 | -0.03361 | negligible | 1.00E+00 | 1.00E+00 | 1.00E+00 |
| bit18 | 0.000963 | 0 | -0.000963 | 3.59E-02 | -0.03113 | negligible | 1.00E+00 | 1.00E+00 | 1.00E+00 |
| bit47 | 0.97676 | 0.97221 | -0.00455 | 4.19E-02 | -0.03019 | negligible | 1.00E+00 | 1.00E+00 | 1.00E+00 |
| bit24 | 0.00182 | 0.000656 | -0.001164 | 6.52E-02 | -0.02735 | negligible | 1.00E+00 | 1.00E+00 | 1.00E+00 |
| bit39 | 0.010288 | 0.012473 | 0.002185 | 1.45E-01 | 0.02164 | negligible | 1.00E+00 | 1.00E+00 | 1.00E+00 |
| bit17 | 0.000766 | 0.000219 | -0.000547 | 1.82E-01 | -0.01982 | negligible | 1.00E+00 | 1.00E+00 | 1.00E+00 |

**Table S7**. Comparison of the performance of selected ligand-based methods, structure-based molecular docking for three molecular targets. Docking to an unrelated protein target (JAK2 kinase) serves as a control. For ligand-based methods, the average values are presented.

|  | **HBV (RNA; Hairpin)** | **PreQ1 (RNA; Pseudoknot)** | **ZTP (RNA; Pseudoknot)** |
| --- | --- | --- | --- |
| **Random Scores** | 0.504 | 0.5 | 0.499 |
| **FP: OpenBabel FP3** | 0.6 | 0.572 | 0.548 |
| **FP: OpenBabel FP4** | 0.557 | 0.538 | 0.54 |
| **Align-It Tanimoto** | 0.512 | 0.534 | 0.545 |
| **Align-It Tversky (DB)** | 0.477 | 0.537 | 0.517 |
| **LISICA 3D** | 0.523 | 0.569 | 0.591 |
| **ShaEP ESP Similarity** | 0.553 | 0.53 | 0.549 |
| **SHAFTS Feature Score** | 0.558 | 0.548 | 0.564 |
| **avg(FP3 LISICA3D)** | 0.593 | 0.586 | 0.705 |
| **avg(FP3 Align-It LISICA3D)** | 0.557 | 0.582 | 0.675 |
| **Docking score (1st pocket)** | 0.479 | 0.537 | 0.597 |
| **Docking Inter score (1st pocket)** | 0.487 | 0.589 | 0.602 |
| **Docking score (2nd pocket)** | 0.532 | 0.509 | 0.546 |
| **Docking Inter score (2nd pocket)** | 0.534 | 0.53 | 0.594 |
| **Docking (best score)** | 0.48 | 0.506 | 0.587 |
| **[control] Docking score** | 0.49 | 0.568 | 0.592 |
| **[control] Docking Inter score** | 0.489 | 0.59 | 0.612 |

**Supplementary Note 2**

To further investigate individual cases of the screening performance we studied two others molecular targets, one with relatively good performance (MYC DNA) and the second with a poor performance (MTOR DNA). See the comments in the main text.

**1. Case study: MYCN DNA.**


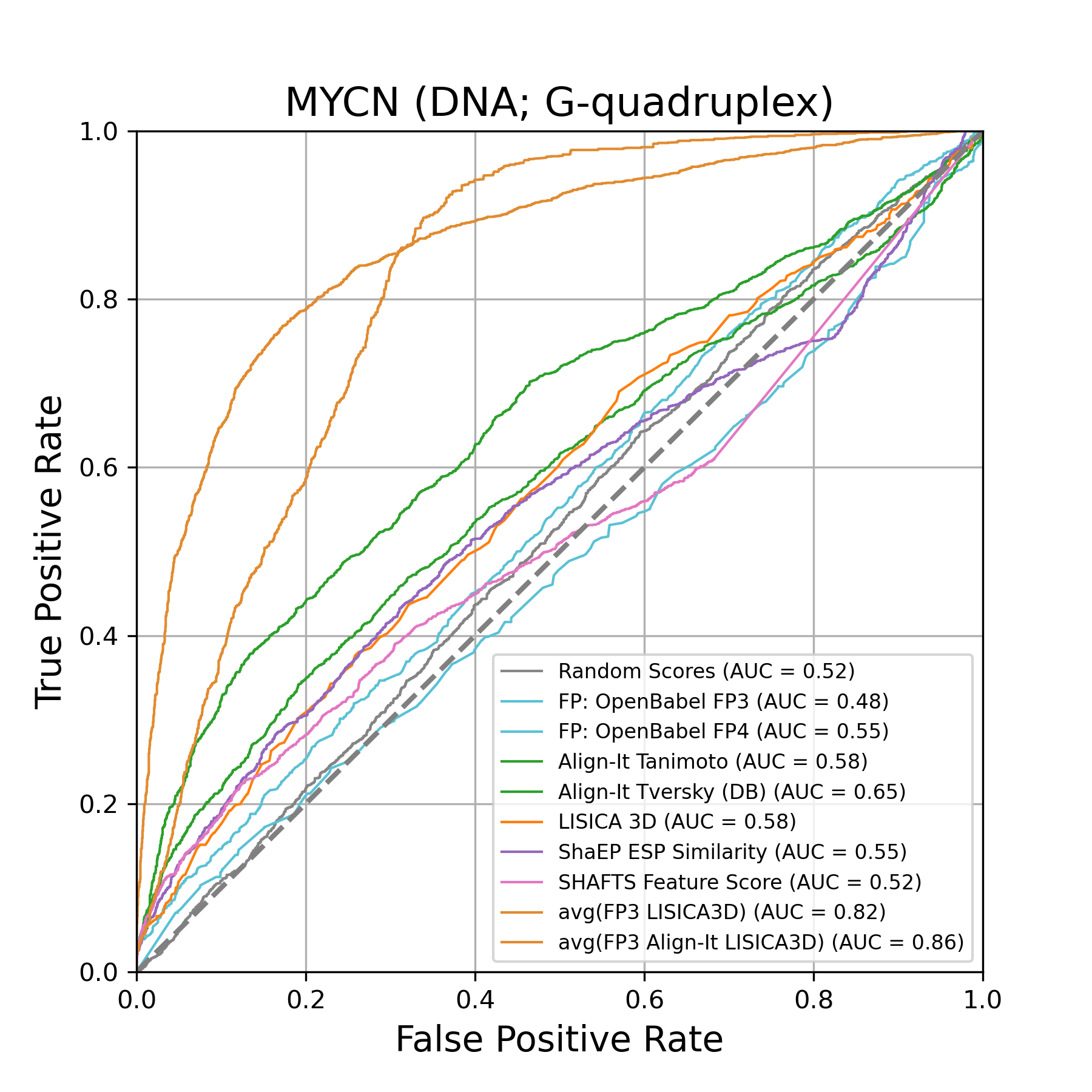


**Fig.** Pooled ROC curves (predicted probabilities and active/inactive labels concatenated from all reference ligands).

**a)** Molecule **1502-1032** as a template. AUROC = 0.911, BEDROC = 0.349, EF1% = 10.192

In top 100 molecules there were three hits (and the template molecule):


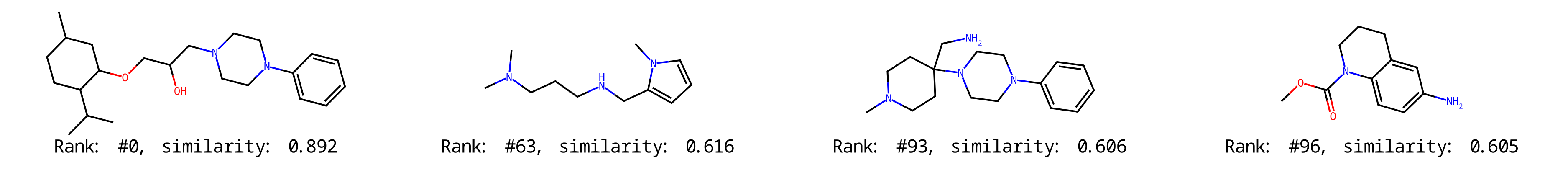


**b)** Molecule **8018-7549** as a template. AUROC = 0.861, BEDROC = 0.388, EF1% = 14.269

In top 100 molecules there were four hits (and the template molecule):


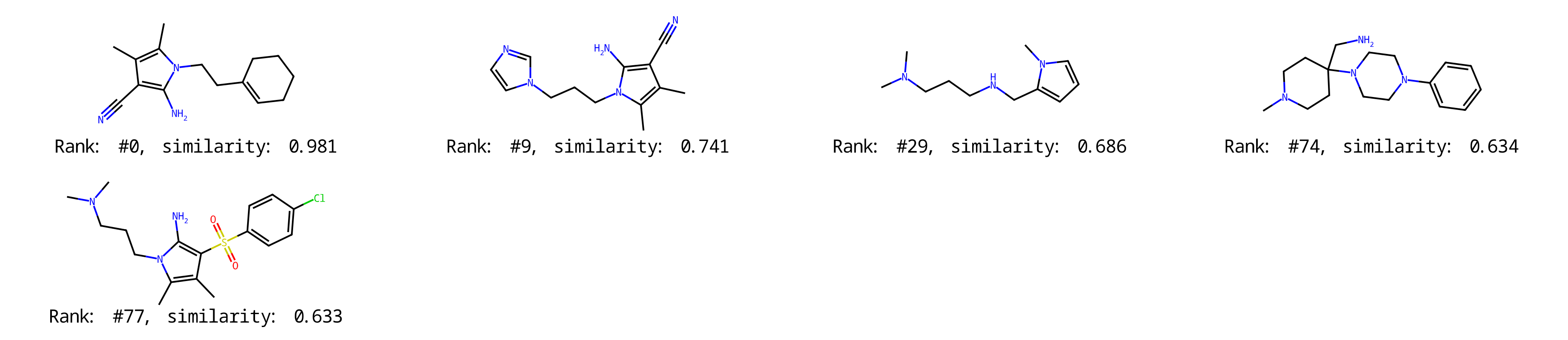


**2. Case study: MTOR DNA**


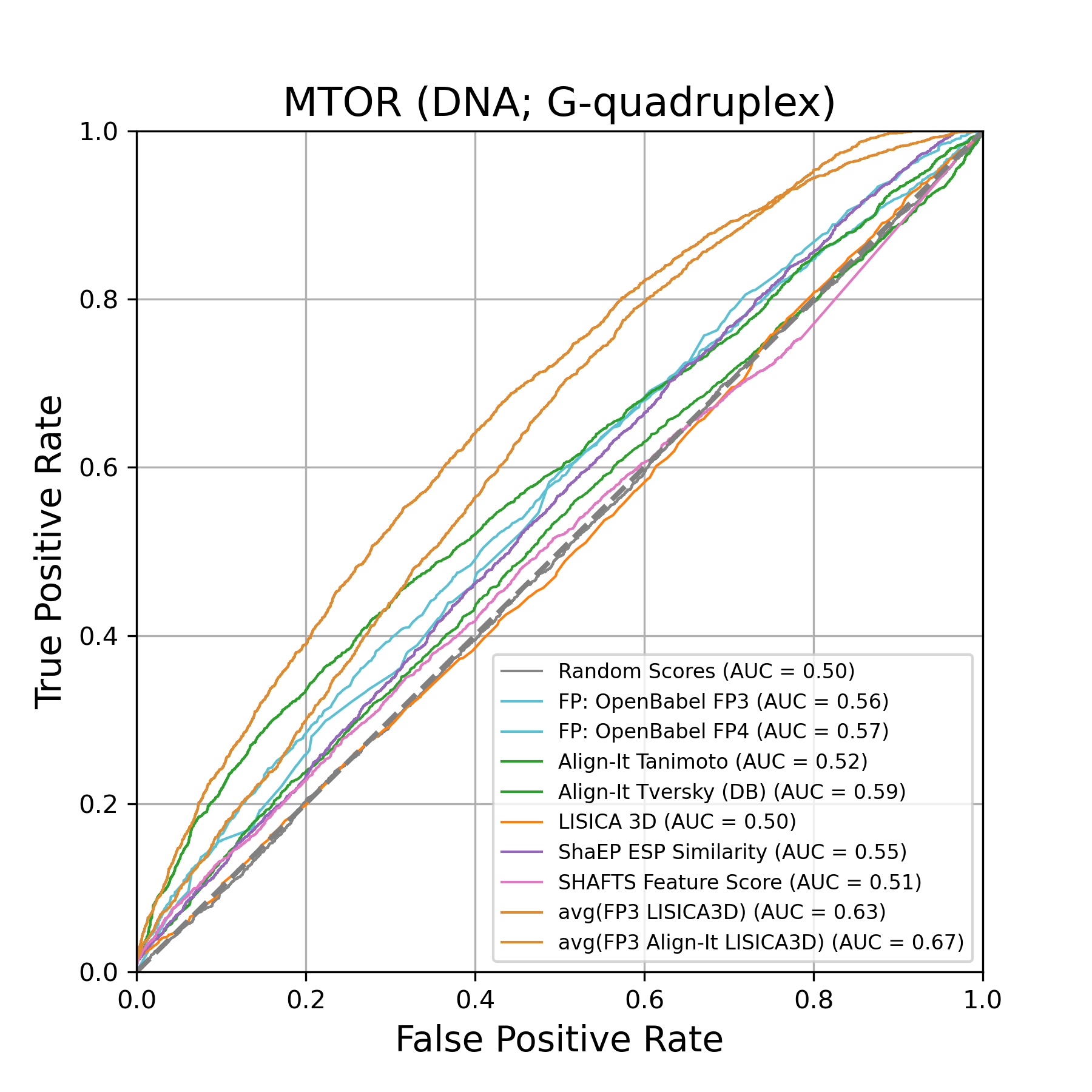


**Fig.** Pooled ROC curves (predicted probabilities and active/inactive labels concatenated from all reference ligands).

**a)** Molecule **20138450** as a template. AUROC = 0.702 BEDROC = 0.186 EF1% = 4.610

In the top ranked 100 molecules there are two hits:


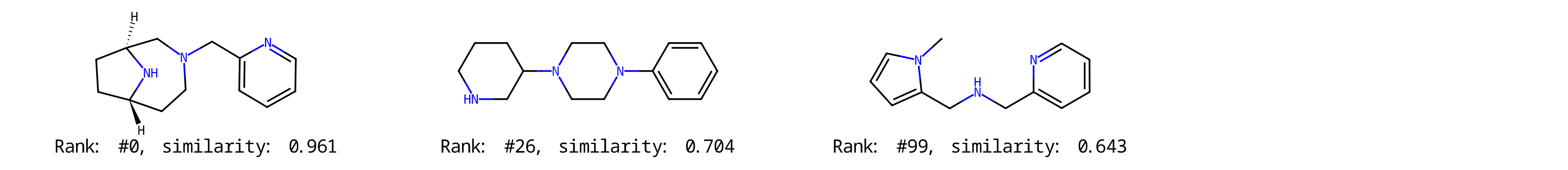


**b)** Molecule **C748-1353** as a template. AUROC = 0.607 BEDROC = 0.141 EF1% = 9.220

In the top ranked 100 there are no hits (apart from the template molecule), in the top 200 ranked molecules there are three hits (and the template).


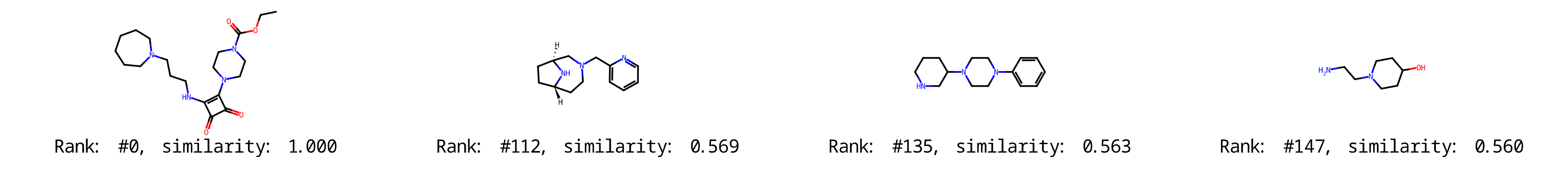


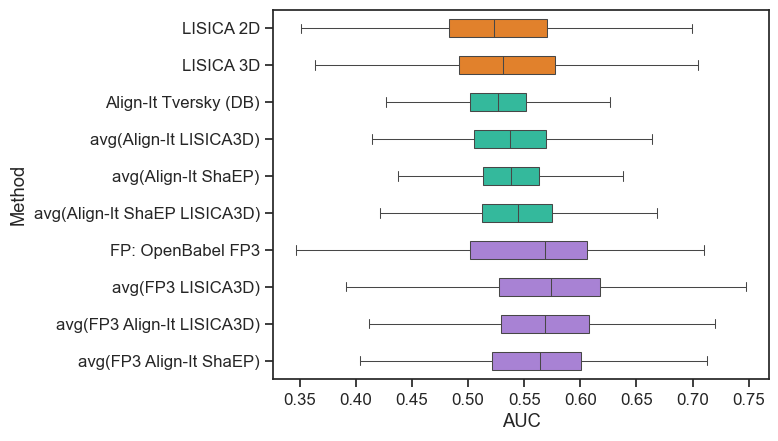
**Fig S6.** Distribution of AUC values for selected methods, showing the role of the inclusion of 3D methods on the performance of consensus scores. Colors determine groups of directly compared methods.

**Table S8**. Mean AUC values for selected methods, showing the role of the inclusion of 3D methods on the performance of consensus scores. Colors determine groups of directly compared methods.

|  | **AUC** | **BEDROC** | **EF1%** | **EF5%** | **EF10%** |
| --- | --- | --- | --- | --- | --- |
| LISICA 2D | 0.529 | 0.082 | 2.881 | 1.589 | 1.370 |
| LISICA 3D | 0.536 | 0.084 | 2.791 | 1.641 | 1.414 |
| Align-It Tversky (DB) | 0.531 | 0.092 | 2.868 | 1.890 | 1.570 |
| avg(Align-It LISICA3D) | 0.541 | 0.098 | 3.318 | 1.983 | 1.638 |
| avg(Align-It ShaEP) | 0.540 | 0.094 | 2.962 | 1.925 | 1.599 |
| avg(Align-It ShaEP LISICA3D) | 0.546 | 0.099 | 3.332 | 2.015 | 1.669 |
| FP: OpenBabel FP3 | 0.551 | 0.117 | 1.642 | 1.370 | 1.297 |
| avg(FP3 LISICA3D) | 0.577 | 0.091 | 2.934 | 1.753 | 1.562 |
| avg(FP3 Align-It LISICA3D) | 0.574 | 0.101 | 3.534 | 2.004 | 1.692 |
| avg(FP3 Align-It ShaEP) | 0.563 | 0.098 | 3.358 | 1.946 | 1.643 |


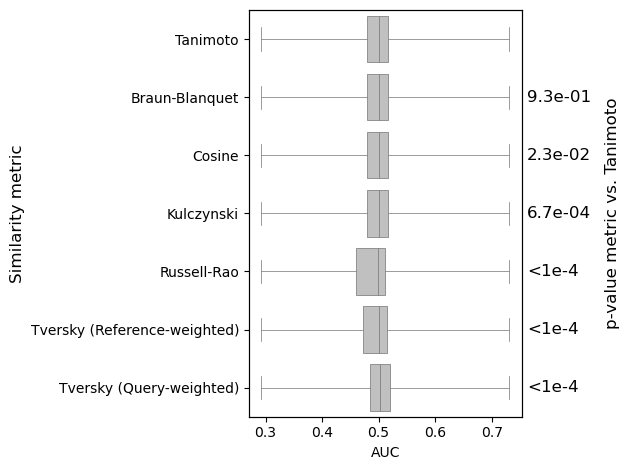
**Fig S7.** Comparison of AUROC values across fingerprint similarity metrics. The statistical significance of pairwise differences relative to the Tanimoto similarity metric is indicated on the right (paired t-tests).
